# Aluminum fluoride-18 labeled folate enables *in vivo* detection of atherosclerotic plaque inflammation by positron emission tomography

**DOI:** 10.1038/s41598-018-27618-4

**Published:** 2018-06-26

**Authors:** Johanna M. U. Silvola, Xiang-Guo Li, Jenni Virta, Päivi Marjamäki, Heidi Liljenbäck, Jarkko P. Hytönen, Miikka Tarkia, Virva Saunavaara, Saija Hurme, Senthil Palani, Harri Hakovirta, Seppo Ylä-Herttuala, Pekka Saukko, Qingshou Chen, Philip S. Low, Juhani Knuuti, Antti Saraste, Anne Roivainen

**Affiliations:** 1 Turku PET Centre, University of Turku, Turku, Finland; 2 Turku PET Centre, Åbo Akademi University, Turku, Finland; 3 Turku Center for Disease Modeling, University of Turku, Turku, Finland; 4 A. I. Virtanen Institute for Molecular Sciences, University of Eastern Finland, Kuopio, Finland; 5 Turku PET Centre, Turku University Hospital, Turku, Finland; 6 Department of Medical Physics, Turku University Hospital, Turku, Finland; 7 Department of Biostatistics, University of Turku, Turku, Finland; 8 Department of Vascular Surgery, Turku University Hospital, Turku, Finland; 9 Science Service Center and Gene Therapy Unit, Kuopio University Hospital, Kuopio, Finland; 10 Department of Pathology and Forensic Medicine, University of Turku, Turku, Finland; 11 Department of Chemistry, Purdue University, West Lafayette, Indiana USA; 12 Heart Center, Turku University Hospital, Turku, Finland; 13 Institute of Clinical Medicine, University of Turku, Turku, Finland

## Abstract

Inflammation plays an important role in the development of atherosclerosis and its complications. Because the folate receptor β (FR-β) is selectively expressed on macrophages, an FR targeted imaging agent could be useful for assessment of atherosclerotic inflammation. We investigated aluminum fluoride-18-labeled 1,4,7-triazacyclononane-1,4,7-triacetic acid conjugated folate (^18^F-FOL) for the detection of atherosclerotic plaque inflammation. We studied atherosclerotic plaques in mice, rabbits, and human tissue samples using ^18^F-FOL positron emission tomography/computed tomography (PET/CT). Compound 2-deoxy-2-[^18^F]fluoro-*D*-glucose (^18^F-FDG) was used as a comparison. Firstly, we found that the *in vitro* binding of ^18^F-FOL co-localized with FR-β-positive macrophages in carotid endarterectomy samples from patients with recent ischemic symptoms. We then demonstrated specific accumulation of intravenously administered ^18^F-FOL in atherosclerotic plaques in mice and rabbits using PET/CT. We noticed that the ^18^F-FOL uptake correlated with the density of macrophages in plaques and provided a target-to-background ratio as high as ^18^F-FDG, but with considerably lower myocardial uptake. Thus, ^18^F-FOL PET/CT targeting of FR-β-positive macrophages presents a promising new tool for the *in vivo* imaging of atherosclerotic inflammation.

## Introduction

Inflammation of the vessel walls plays a role in the development of atherosclerosis and its complications^1,2^. Positron emission tomography/computed tomography (PET/CT) imaging with glucose analog 2-deoxy-2-[^18^F]fluoro-*D*-glucose (^18^F-FDG) has been used for imaging inflammation in atherosclerosis on the basis of the high glucose consumption of macrophages in inflamed plaques^3,4^. However, detection of vascular inflammation is challenging because of the small size of the target and the non-specific nature of ^18^F-FDG^5^.

Several alternative approaches for imaging inflammatory activity in atherosclerosis have been tested, such as targeting somatostatin or mannose receptors, choline metabolism, or translocator protein expression; however, none has so far proven to be superior to ^18^F-FDG^3^. Since folate receptor β (FR-β) is highly expressed on macrophages^6,7^, a series of folate-based tracers have been evaluated for imaging of inflammatory disorders^8–12^. Previously, folate-based ^99m^Tc-EC20 and ^111^In-EC0800 have shown potential for single-photon emission computed tomography (SPECT) imaging of atherosclerosis in mice^8,9^. However, it is not known whether an FR targeted tracer would provide a sufficient target-to-background ratio (TBR) for *in vivo* PET/CT imaging of inflammation in atherosclerosis.

Accordingly, we synthesized aluminum fluoride-18-labeled 1,4,7-triazacyclononane-1,4,7-triacetic acid conjugated folate ([^18^F]AlF-NOTA-folate or ^18^F-FOL) with high FR binding affinity (dissociation constant K_d_ ~ 1.0 nM)^13^ and high specific radioactivity. We hypothesized that rapid blood clearance combined with high uptake in FR-β-positive macrophages would result in sufficient TBR for *in vivo* detection of inflamed atherosclerotic plaques with ^18^F-FOL PET/CT.

To test the hypothesis, we first evaluated the binding of ^18^F-FOL to FR-β-positive macrophages in carotid endarterectomy sections from patients with recent ischemic symptoms. Next, we investigated the uptake and specificity of intravenously (i.v.) administered ^18^F-FOL for detection of atherosclerotic plaques in murine and rabbit models of atherosclerosis. As a final point, we compared *in vivo* ^18^F-FOL and ^18^F-FDG PET/CT imaging of inflamed lesions in mice and rabbits with atherosclerosis.

## Results

### Generation of folate-based PET imaging agent

**Figure 1:**
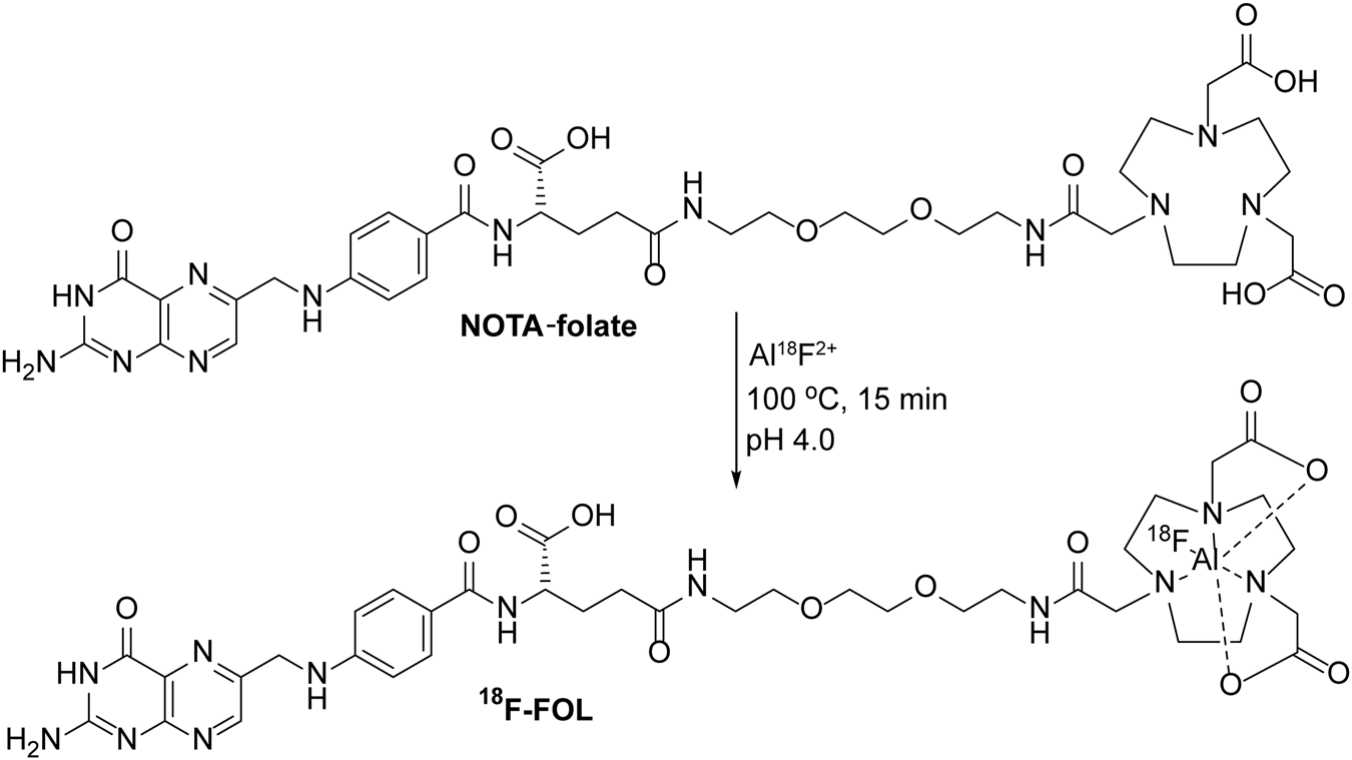
Radiosynthesis of ^18^F-FOL. In the structure of ^18^F-FOL (molecular formula C_37_H_50_^18^FN_12_O_12_, calculated molecular weight 899.8615), the folic acid moiety is 424.3970 Da. The linker, chelator, and Al^18^F moieties are 475.4645 Da in total, and account for 53% of the molecular weight of ^18^F-FOL.

By incubating [^18^F]-fluoride with aluminum chloride (AlCl_3_) followed by heating in the presence of NOTA-folate at 100 °C, we produced ^18^F-FOL (Fig. 1) with a high molar activity (77 ± 22 GBq/µmol) and radiochemical purity (≥95% at the end of synthesis). High-performance liquid chromatography (HPLC) analysis of ^18^F-FOL in phosphate-buffered saline (PBS; pH 7.4) containing 8% ethanol and 4–7% polypropylene glycol at room temperature, at up to 4 hours after radiosynthesis, did not result in the detection of radiolysis products. The distribution coefficient (Log *D*, *n*-octanol/PBS, pH 7.4) of −3.3 ± 0.44 indicated high hydrophilicity. The plasma protein binding of i.v. injected ^18^F-FOL was 16% ± 6.4% in mice and 15% ± 9.5% in rabbits. Up to 60 minutes post-injection, the count from the intact tracer in mice plasma was still 85% ± 6.0% of the total radioactivity, thereby indicating good *in vivo* stability.

### ^18^F-FOL binding co-localizes with FR-β positive macrophages in human carotid endarterectomy sections *in vitro*

**Figure 2:**
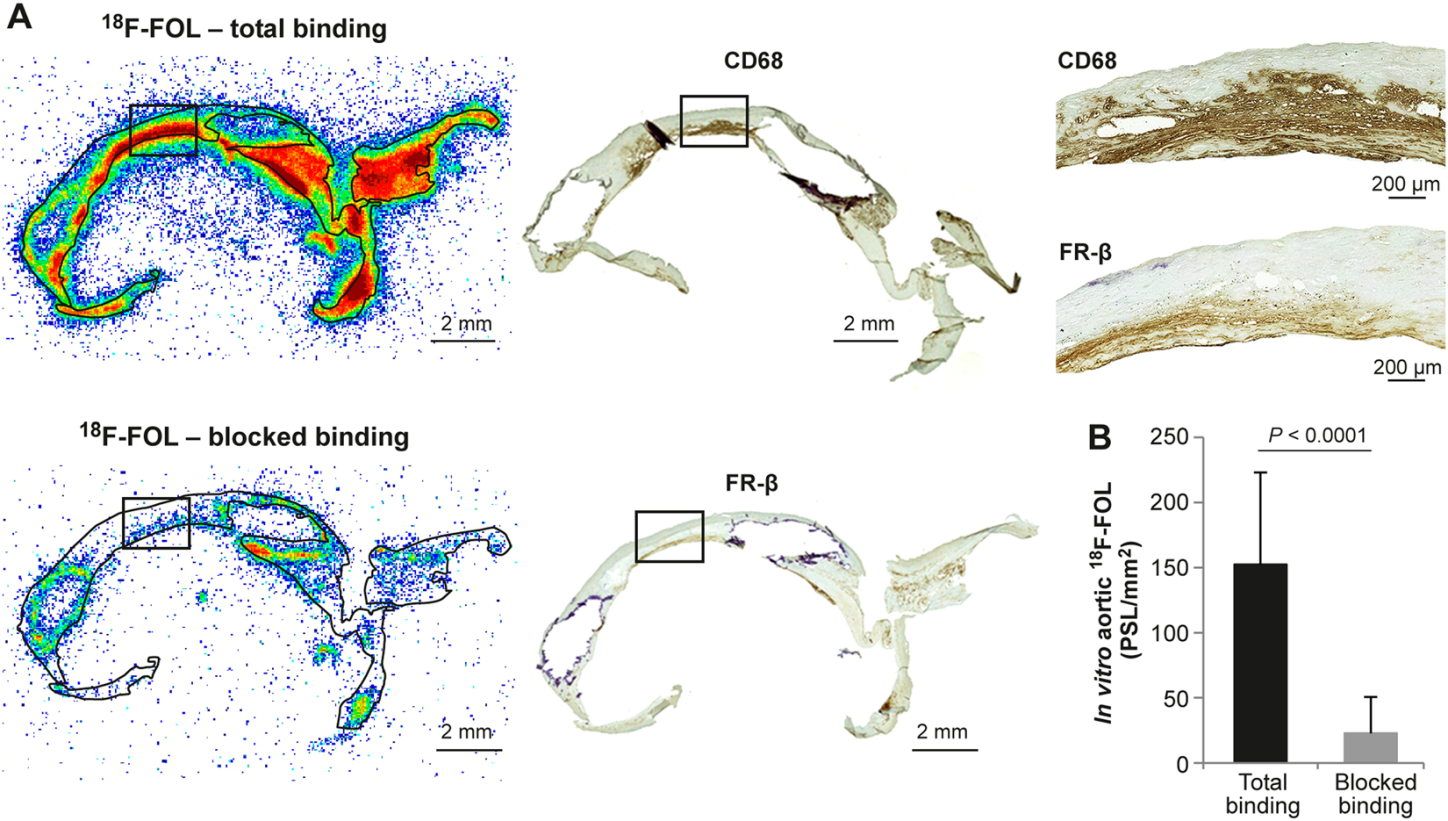
*In vitro* binding of ^18^F-FOL in human carotid artery samples. ^18^F-FOL autoradiography and immunohistochemistry of adjacent carotid endarterectomy samples from patients with recent ischemic symptoms (*n* = 5). (**A**) Left: Representative autoradiographs of total and folate glucosamine-blocked binding of ^18^F-FOL on 7 µm adjacent cryosections. Middle: CD68 (macrophages) and folate receptor β (FR-β) immunohistochemical staining of the same cryosections as in figure A. Note that the other section has ruptured during the CD68 staining process. Top right: high-power views of CD68 and FR-β immunohistochemical staining in the area within the black rectangle on the autoradiographs. CD68 and FR-β positive cells appear as a brown color. (**B**) *In vitro* autoradiography-based quantification of ^18^F-FOL binding in tissue cryosections verifies the signal’s specificity to folate receptors (paired *t-*test). Error bars denote standard deviation. PSL/mm^2^ = photostimulated luminescence per mm^2^.

We obtained carotid endarterectomy samples from five patients with recent transient ischemic attack and severe (≥80%) stenosis of the carotid artery, incubated cryosections with ^18^F-FOL *in vitro*, and then detected its binding with high-resolution digital autoradiography. Following this, we immunohistochemically stained the same sections and observed co-localization of ^18^F-FOL binding, and macrophages positive to FR-β and cluster of differentiation 68 (CD68; Fig. 2A). The quantitative *in vitro* autoradiography analysis revealed significantly lower ^18^F-FOL binding (88% ± 8.9% reduction) in sections incubated with folate glucosamine (blocked binding) than with the total ^18^F-FOL binding without blocking (Fig. 2B), thus indicating that tracer binding in lesions was specific to FR.

In addition, we cultured the purified monocytes from healthy human blood under M1-polarising (interferon γ [IFN-γ] and lipopolysaccharides [LPS]) or M2-polarising (macrophage colony-stimulating factor [M-CSF], interleukin 4 [IL-4] and IL-10) conditions and evaluated the binding of ^18^F-FOL to macrophages during *in vitro* differentiation. We found that ^18^F-FOL binds to both M1 and M2 macrophages although clearly more to M2-polarized macrophages (Fig. S1).

### Intravenously administered ^18^F-FOL accumulates in inflamed atherosclerotic lesions in mice

We used 6-month-old, high-fat fed, low-density lipoprotein receptor deficient, male mice, which expressed only apolipoprotein B100 (LDLR^−/−^ApoB^100/100^), as an atherosclerosis model. Regular chow-fed 9-month-old C57BL/6N male mice were used as healthy controls (Table 1). We evaluated the biodistribution and kinetics of i.v. administered ^18^F-FOL in atherosclerotic and healthy control mice using PET/CT imaging, gamma counting, and digital autoradiography. A blocking study with co-injection of a 100-fold excess of folate glucosamine was performed to clarify tracer uptake specificity.

**Table 1:** Basic characteristics of investigated animals.

	LDLR^−/−^ApoB^100/100^ atherosclerotic mice	C57BL/6N control mice	Watanabe atherosclerotic rabbits
Age, months	6	9	45
High-fat diet, months	4	ND	ND
Female/male animals, no.	0/12	0/9	1/3
Weight, g*	37 ± 5	38 ± 6	3200 ± 800
*In vivo* blocking study, no.	3	ND	ND
*In vivo* ^18^F-FOL PET/CT, no.	9 + 2^†^	6	4
*In vivo* ^18^F-FDG PET/CT, no.	8	4	4
*Ex vivo* ^18^F-FOL gamma counting, no.	9 + 3^†^	9	4
*Ex vivo* ^18^F-FOL autoradiography, no.	8 + 2^†^	6	4
*En face* aorta ^18^F-FOL PET, no.	1 + 1^†^	1	ND

LDLR^−/−^ApoB^100/100^ = low-density lipoprotein receptor deficient mouse expressing only apolipoprotein B100; ND = not done; Blocking study = *in vivo* competition assay with 100-fold molar excess of folate glucosamine injected 1 min before ^18^F-FOL; no = number of investigated animals.

*Values are mean ± SD.

^†^No. for blocking study.

**Figure 3:**
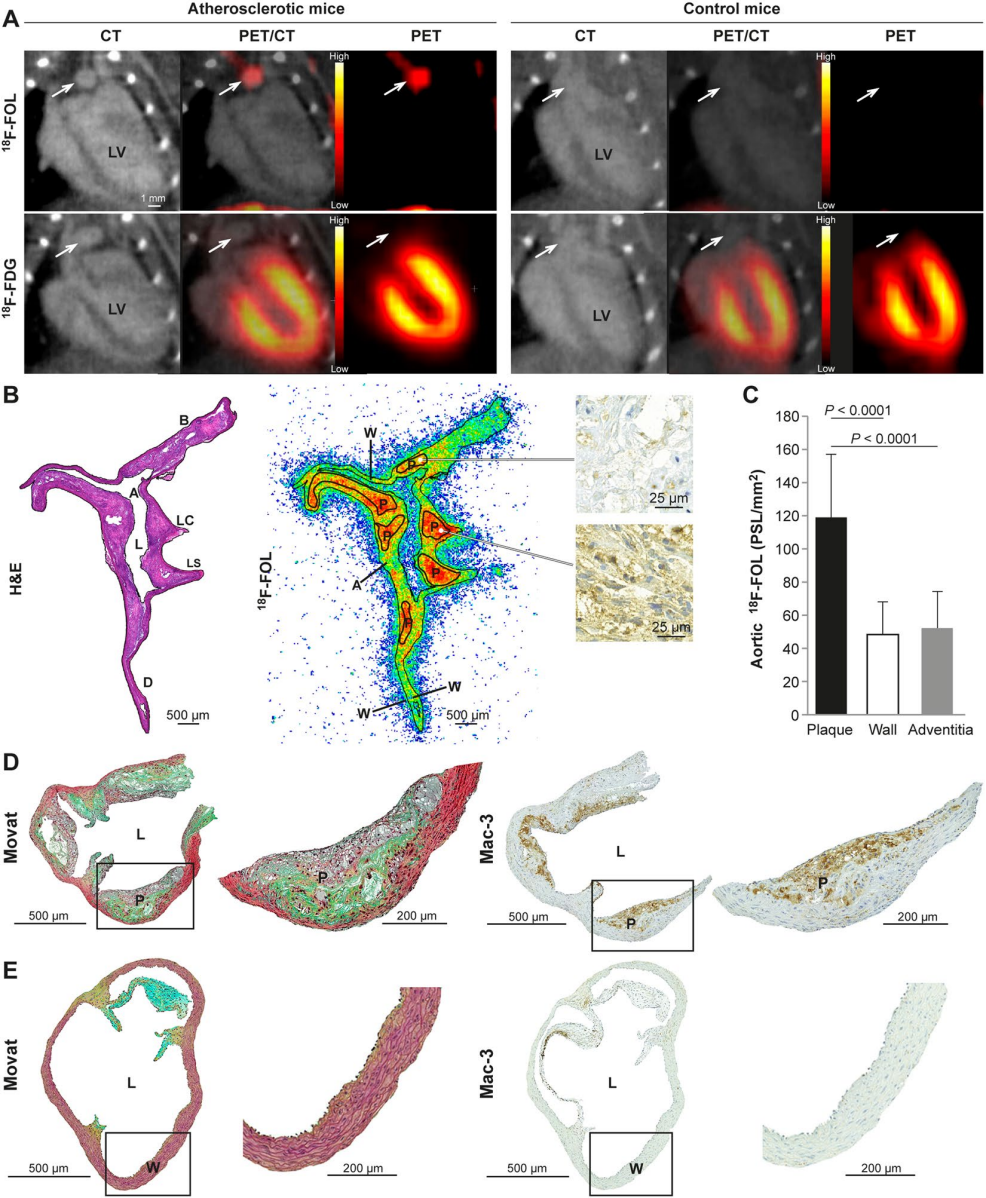
Overview of the mouse studies. (**A**) Representative coronal views of *in vivo* ^18^F-FOL and ^18^F-FDG PET/CT images (60–90 min post-injection) of atherosclerotic LDLR^−/−^ApoB^100/100^ and healthy C57BL/6N control mice. White arrows denote the aortic arch. LV = left ventricle. PET images are displayed in the same color scale. (**B**) Left: hematoxylin-eosin (H&E) staining of a longitudinally sectioned atherosclerotic mouse aorta. A = arch; AA = ascending aorta; B = brachiocephalic artery; D = descending thoracic aorta; L = lumen; LC = left common carotid artery; LS = left subclavian artery. Middle: superimposed ^18^F-FOL *ex vivo* autoradiograph and H&E staining, with black lines representing the tissue contour and regions of interest. P = plaques (excluding media); W = healthy vessel wall (no lesion formation); A = adventitia (mainly adipose tissue around the aorta). Right: Mac-3 immunohistochemical staining corresponding to a plaque with low (top) or high (bottom) ^18^F-FOL uptake. (**C**) Quantification of ^18^F-FOL binding on the *ex vivo* autoradiography of atherosclerotic mice aorta (*n* = 8). *P* value, one-way ANOVA with Tukey’s correction. PSL/mm^2^ = photostimulated luminescence per mm^2^ normalized for injected radioactivity dose. Error bars denote standard deviation. (**D**) The aortas of LDLR^−/−^ApoB^100/100^ mice contained atherosclerotic plaques that were mostly of the fibroatheroma-type with a well-defined fibrous cap whereas (**E**) the aortas of healthy C57BL/6N control mice showed no signs of atherosclerosis. Representative formalin-fixed, paraffin-embedded aortic root sections were stained with Movat’s pentachrome (black = nuclei; yellow = collagen, reticular fibers; blue = ground substance, mucin; bright red = fibrin; red = muscle) or with anti-mouse Mac-3 immunohistochemistry (brown color). The high-power views are of the area within the black rectangle on the left images. Mac-3 positive cells (macrophages) appear brown in color. L = lumen; P = plaque; W = healthy vessel wall.

We performed *in vivo* ^18^F-FOL PET/CT, which showed that the highest maximum TBR (maximum standardized uptake value (SUV) of aortic arch divided by mean SUV of blood measured from the vena cava; SUV_max, aortic arch_/SUV_mean, blood_) of 1.5 ± 0.34 occurred in atherosclerotic mice 60–90 min after ^18^F-FOL injection. For comparison, the maximum TBR in aortic arch of healthy control mice was significantly lower at 0.71 ± 0.18 (*P* = 0.00019; Figs 3A and S2). A comparison revealed that the myocardial uptake of ^18^F-FDG was significantly higher than that of ^18^F-FOL (SUV_max,60–90 min_ 12 ± 4.3 *vs*. 0.36 ± 0.069, *P* < 0.0001, respectively), which hampered the visualization and quantification of ^18^F-FDG uptake in mice aortic arches (Fig. 3A). Blood radioactivity concentrations (SUV_mean, 60–90 min_) of LDLR^−/−^ApoB^100/100^ mice were 0.39 ± 0.15 and 0.68 ± 0.22 (*P* = 0.0057) for ^18^F-FOL and ^18^F-FDG, respectively.

Similarly, according to *ex vivo* gamma counting, the percentage of injected radioactivity dose per gram of aorta (%ID/g) was significantly higher in atherosclerotic mice (2.4 ± 0.56%ID/g at 120 min post-injection) than in healthy controls (1.3 ± 0.46%ID/g, *P* = 0.00062) or atherosclerotic mice from the blocking study (0.28 ± 0.15%ID/g, *P* = 0.0040). Additionally, the radioactivity concentration in atherosclerotic aorta was >5-fold that in blood (0.48 ± 0.20%ID/g, *P* < 0.0001). In both mouse strains, the highest radioactivity was observed in FR-positive kidneys. Excess radioactivity was primarily excreted in the urine, and uptake in liver, heart and other organs was significantly lower than in the kidneys (Table 2).

**Table 2:** *Ex vivo* biodistribution of ^18^F-FOL in mice and rabbits.

Tissue	LDLR^−/−^ApoB^100/100^ atherosclerotic mice (*n* = 9)	LDLR^−/−^ApoB^100/100^ mice + blocking (*n* = 3)	C57BL/6N control mice (*n* = 9)	Watanabe atherosclerotic rabbits (*n* = 4)
Aorta	2.4 ± 0.56	0.28 ± 0.15 (*P* = 0.0040)*	1.3 ± 0.46 (*P* = 0.00062)^†^	0.075 ± 0.026^§^ 0.048 ± 0.018^║^
BAT	0.89 ± 0.43	0.10 ± 0.024 (*P* = 0.012)*	0.50 ± 0.37 (*P* = 0.084)^†^	ND
Blood	0.48 ± 0.20	0.14 ± 0.059 (*P* = 0.0037)*	0.20 ± 0.065 (*P* = 0.0010)^†^	0.0056 ± 0.0027
Heart	0.66 ± 0.17	0.080 ± 0.032 (*P* < 0.0001)*	0.46 ± 0.089 (*P* = 0.0068)^†^	0.095 ± 0.086
Kidneys	110 ± 28	9.3 ± 0.63 (*P* < 0.0001)*	104 ± 18 (*P* = 0.77)^†^	0.92 ± 0.34
Lungs	1.2 ± 0.37	0.19 ± 0.063 (*P* < 0.0001)*	0.54 ± 0.15 (*P* = 0.00023)^†^	0.032 ± 0.012
Liver	2.8 ± 1.3	0.98 ± 0.45 (*P* = 0.052)*	1.4 ± 1.1 (*P* = 0.031)^†^	0.55 ± 0.69
Muscle	0.52 ± 0.10	0.059 ± 0.012 (*P* = 0.0014)*	0.43 ± 0.23 (*P* = 0.44)^†^	0.015 ± 0.0041
Pancreas	1.2 ± 0.38	0.15 ± 0.037 (*P* < 0.0001)*	0.64 ± 0.21 (*P* = 0.0014)^†^	0.15 ± 0.032
Plasma	0.70 ± 0.18	0.20 ± 0.10 (*P* < 0.0001)*	0.30 ± 0.11 (*P* < 0.0001)^†^	0.14 ± 0.16
Small intestine	4.2 ± 2.8	4.6 ± 0.85 (*P* = 0.99)*	3.1 ± 1.8 (*P* = 0.70)^†^	0.20 ± 0.066
Spleen	0.34 ± 0.072	0.13 ± 0.043 (*P* = 0.079)*	0.29 ± 0.20 (*P* = 0.74)†	0.75 ± 0.53
Thymus	1.7 ± 1.0	0.20 ± 0.11 (*P* = 0.012)*	0.67 ± 0.28 (*P* = 0.013)^†^	ND
Urine	66 ± 48	261 ± 97 (*P* = 0.0013)*	84 ± 51 (*P* = 0.50)^†^	0.15 ± 0.20
WAT	0.46 ± 0.16	0.058 ± 0.029 (*P* = 0.0020)*	0.38 ± 0.16 (*P* = 0.49)^†^	0.027 ± 0.023
Aorta-to-blood	5.2 ± 1.5 (*P* < 0.0001)^‡^	2.5 ± 1.8 (*P* = 0.36)^‡^	6.8 ± 2.6 (*P* < 0.0001)^‡^	16 ± 10^§^ (*P* = 0.013)^‡^
Aorta-to-heart	3.6 ± 0.76 (*P* < 0.0001)^‡^	2.5 ± 1.8 (*P* = 0.11)^‡^	2.9 ± 0.74 (*P* < 0.0001)^‡^	1.3 ± 1.0^§^ (*P* = 0.67)^‡^
Aorta-to-muscle	2.3 ± 0.52 (*P* < 0.0001)^‡^	0.28 ± 0.059 (*P* = 0.13)^‡^	1.3 ± 0.42 (*P* = 0.00015)^‡^	5.6 ± 2.7^§^ (*P* = 0.21)^‡^

Results are expressed as a percentage of injected radioactivity dose per gram of tissue (mean ± SD with two significant figures) and obtained at 120 min post-injection. The blocking study was performed by injecting a 100-fold molar excess of folate glucosamine 1 min previous to the ^18^F-FOL injection.

*Difference between LDLR^−/−^ApoB^100/100^ atherosclerotic mice *vs*. atherosclerotic mice + blocking, as assessed by independent samples *t*-test.

^†^Difference between LDLR^−/−^ApoB^100/100^ atherosclerotic *vs*. C57BL/6N control mice as assessed by independent samples *t*-test.

^‡^Intra-animal comparison assessed by paired *t*-test.

^§^Aorta with advanced atherosclerosis.

^║^Aorta with mild atherosclerosis.

BAT = brown adipose tissue; WAT = white adipose tissue; ND = not determined.

*Ex vivo* PET imaging of excised aortas 120 minutes after ^18^F-FOL injection followed by *en face* Oil-Red-O staining revealed the highest tracer uptake in the most prominent atherosclerotic lesions, i.e., in the aortic arch of the LDLR^−/−^ApoB^100/100^ mice (Fig. S3).

**Figure 4:**
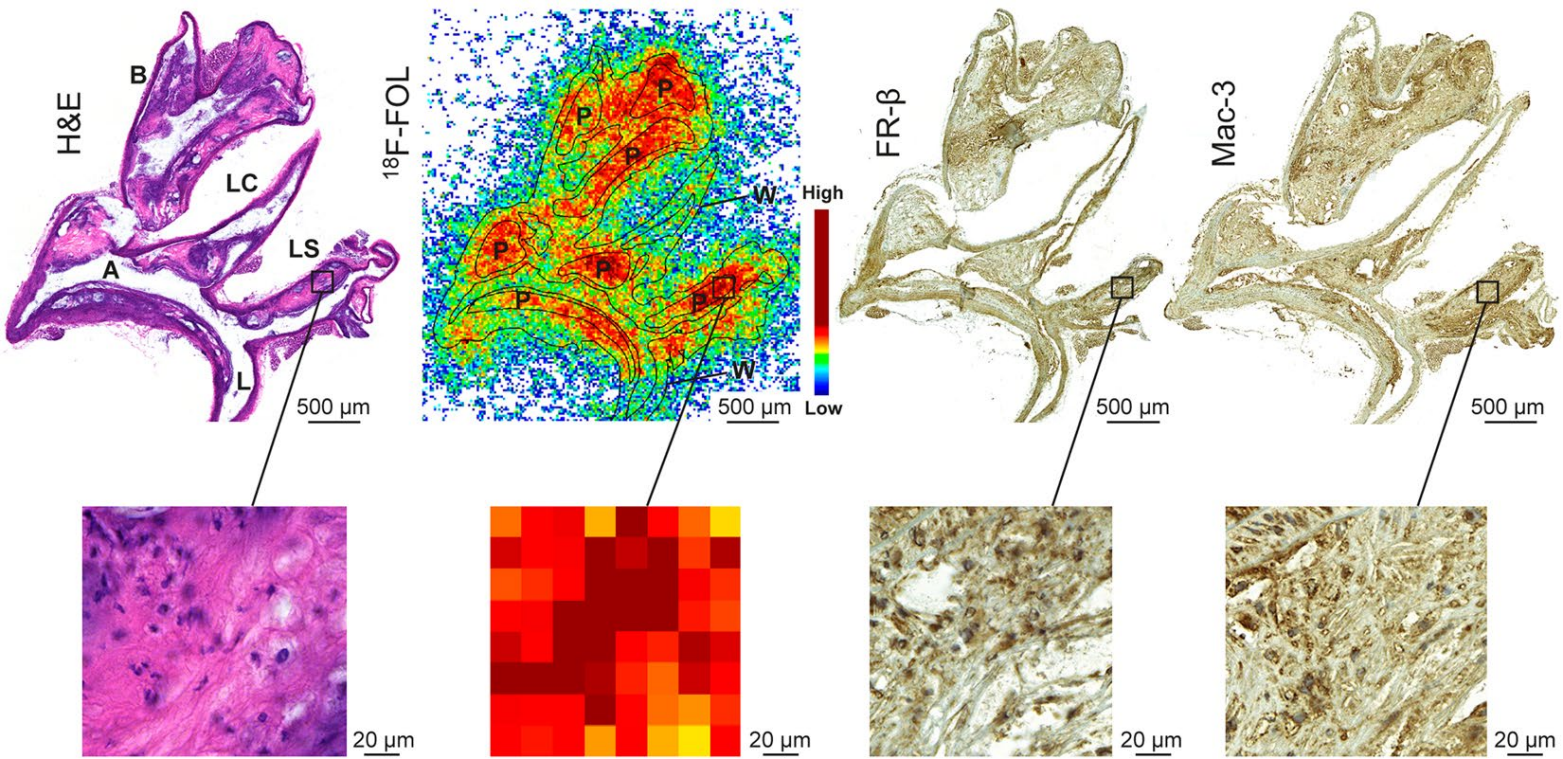
*Ex vivo* ^18^F-FOL autoradiography, histology and immunohistochemistry of mouse aorta. Representative hematoxylin-eosin (H&E), ^18^F-FOL *ex vivo* autoradiograph, FR-β and Mac-3 immunohistochemical staining (macrophages) of adjacent aorta cryosections. The images on the bottom show high-power views of the area within the black rectangle on the upper images. A = arch; B = brachiocephalic artery; L = lumen; LC = left common carotid artery; LS = left subclavian artery. P = plaque; W = healthy vessel wall.

When the ^18^F-FOL uptake in mouse aortas was explored in more detail using autoradiography and immunohistochemistry of tissue cryosections, we noticed that the ^18^F-radioactivity was co-localized with Mac-3- and FR- β-positive macrophage-rich plaques (Figs 3B and 4). The evaluation of count densities (photostimulated luminescence per square millimeter, PSL/mm^2^) revealed a significantly higher uptake of ^18^F-FOL in mouse atherosclerotic plaques than occurred in healthy vessel walls (plaque-to-healthy vessel wall ratio 2.6 ± 0.58, *P* < 0.0001) or adventitia (plaque-to-adventitia ratio 2.4 ± 0.55, *P* < 0.0001; Fig. 3C). Furthermore, we determined similar healthy vessel wall-to-adventitia ratios in atherosclerotic and control mice (0.97 ± 0.27 *vs*. 1.0 ± 0.12, respectively, *P* = 0.73).

In the blocking study, i.v. co-injection of ^18^F-FOL and 100-fold excess of folate glucosamine reduced average plaque uptake by 92%, as determined by *ex vivo* autoradiography of aorta cryosections. When blocking was performed in aorta cryosections *in vitro*, it reduced total binding of ^18^F-FOL by 89% ± 3.5%.

According to modified Movat’s pentachrome and anti-Mac-3 staining, the aortas of the LDLR^−/−^ApoB^100/100^ mice contained large and inflamed atherosclerotic plaques, which were mostly of the fibroatheroma-type, with a well-defined fibrous cap. The intima-to-media ratio was 3.7 ± 2.1, and the lesions were abundantly infiltrated with Mac-3-positive macrophages (13% ± 4.6% of the neointimal area), which were situated mostly in the fibrous cap (Fig. 3D). The control mice showed no signs of atherosclerosis (Fig. 3E). The atherosclerotic plaques showed positivity for anti-inducible nitric oxide synthase (iNOS) and anti-mannose receptor C-type 1 (MRC-1), indicating M1 and M2 polarized macrophages, respectively (Fig. S4).

### *In vivo* [^18^F]FOL PET/CT detects atherosclerotic lesions in rabbit aorta

We next used Watanabe heritable hyperlipidemic rabbits, with subdiaphragmatic denudation in the abdominal aorta, as another atherosclerosis model. The rabbits were 45-months-old and fed on regular chow, and three males and one female were used.

**Figure 5:**
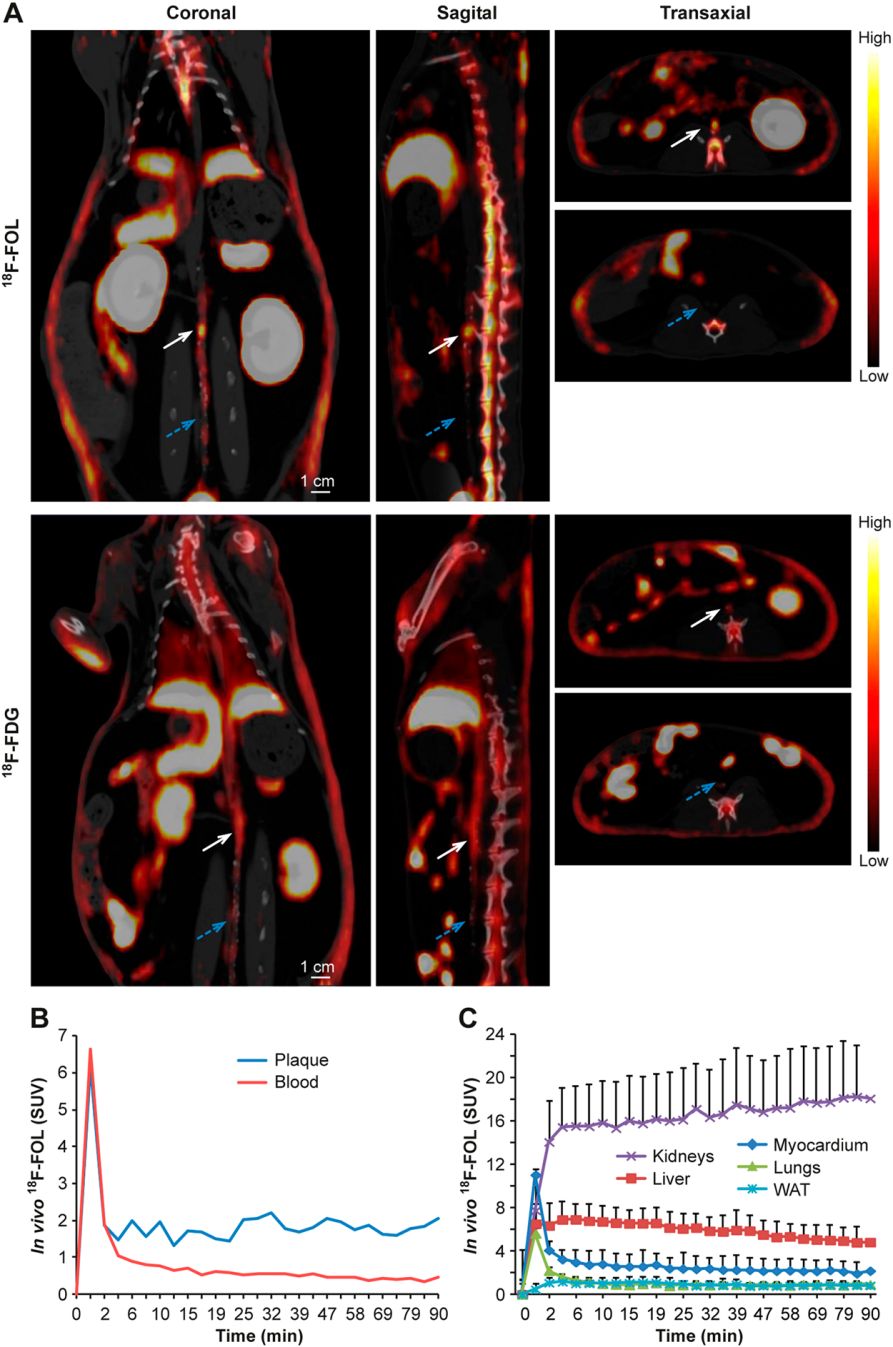
^18^F-FOL PET/CT imaging of rabbits. (**A**) Representative coronal views of *in vivo* PET/CT images of a rabbit (3.4 kg, tracer doses 40 MBq/kg) with ^18^F-FOL (88–90 min post-injection) and ^18^F-FDG (170–180 min post-injection). White arrows denote a segment with advanced atherosclerosis in the abdominal aorta, with an ^18^F-FOL maximum target-to-background ratio (TBR = maximum standardized uptake value (SUV) of aorta divided by mean SUV of blood as determined from the inferior vena cava, SUV_max, aorta_/SUV_mean, blood_) of 6.0 and an ^18^F-FDG maximum TBR of 2.4. Enlarged coronal PET/CT images of the aorta are shown in the right panels. Blue arrows denote aorta with lower tracer uptake. PET images are displayed in the same color scale. (**B**) Representative ^18^F-FOL time-activity curves of plaque (white arrow in Figure A) and blood. (**C**) Distribution kinetics of ^18^F-FOL in the selected organs (mean ± SD, *n* = 4).

**Figure 6:**
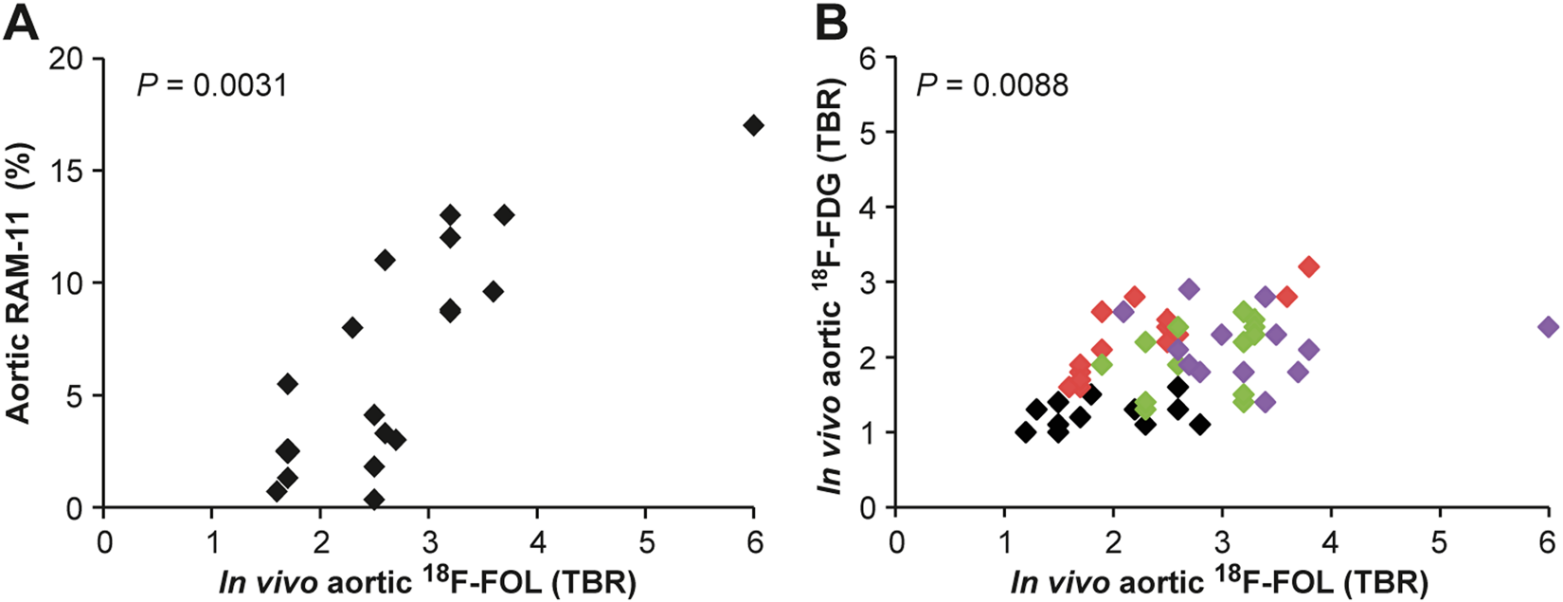
^18^F-FOL and ^18^F-FDG uptake, and density of macrophages in rabbit aorta. Association between *in vivo* ^18^F-FOL uptake in the rabbit aorta and (**A**) RAM-11-positive (macrophages) area (%; linear mixed model; slope = 3.82, 95% confidence interval of 1.60 to 6.05), and (**B**) ^18^F-FDG uptake (linear mixed model; slope = 0.22, 95% confidence interval of 0.058 to 0.38). The data of each rabbit are displayed in a different color. TBR = target-to-background ratio; maximum standardized uptake value (SUV) of aorta divided by mean SUV of blood as determined from the inferior vena cava (SUV_max, aorta_/SUV_mean, blood_).

We performed a direct comparison of ^18^F-FOL and ^18^F-FDG *in vivo* PET/CT imaging of atherosclerotic aorta in Watanabe rabbits. Our results revealed a rapid clearance of ^18^F-FOL from circulating blood, and a comparable maximum TBR (SUV_max, aorta_/SUV_mean, blood_) in the atherosclerotic aorta to that of ^18^F-FDG (Fig. 5). There was a high association between the maximum TBR of ^18^F-FOL PET and the areal percentage of RAM-11-positive staining of macrophages (linear mixed model *P* = 0.0031) in the corresponding segment of the aorta (Fig. 6A). Furthermore, there was an association between maximum TBR obtained with ^18^F-FOL and ^18^F-FDG PET (linear mixed model *P* = 0.0088) in the same segments of the aorta (Fig. 6B). The average uptake of ^18^F-FOL (2.6) in the whole aorta was similar to that of ^18^F-FDG (1.9; *P* = 0.074; Table 3).

**Table 3:** Quantification of ^18^F-FOL and ^18^F-FDG PET/CT in rabbit aorta.

	Global	Macrophage density*			Histopathology^†^		
		Highest	Lowest	*P* ^‡^	Advanced	Mild	*P* ^§^
RAM-11 (%)	7.8 ± 3.2^║^	14 ± 3.1^║^	3.8 ± 3.9^║^	0.064^║^	9.5 ± 4.6^║^	2.2 ± 1.1**	0.31^║^
^18^F-FOL (TBR^¶^)	2.6 ± 0.63	3.9 ± 1.8^║^	2.5 ± 0.20^║^	0.27^║^	2.7 ± 0.79	1.9 ± 0.47	0.023
^18^F-FDG (TBR^¶^)	1.9 ± 0.45	2.4 ± 0.15^║^	1.9 ± 0.55^║^	0.30^║^	1.8 ± 0.44	1.8 ± 0.38	0.76
*P* ^#^	0.074	0.29^║^	0.15^║^		0.072	0.35	

Results are mean ± SD (*n* = 4 rabbits, except ^║^*n* = 3 and **n = 2). All *P* values, paired *t*-test.

*Based on anti-RAM-11 immunohistochemistry.

^†^Based on hematoxylin-eosin staining.

^‡^Highest *vs*. Lowest.

^§^Advanced *vs*. Mild.

^¶^Target-to-background ratio = SUV_max, aorta_/SUV_mean. blood_.

^#18^F-FOL *vs*. ^18^F-FDG.

*Ex vivo* gamma counts of excised tissue samples were in accordance with the *in vivo* ^18^F-FOL PET imaging results. Compared with atherosclerotic mice, our 45-month-old rabbits showed the highest ^18^F-FOL uptake in the kidneys, spleen and liver (Table 2).

**Figure 7:**
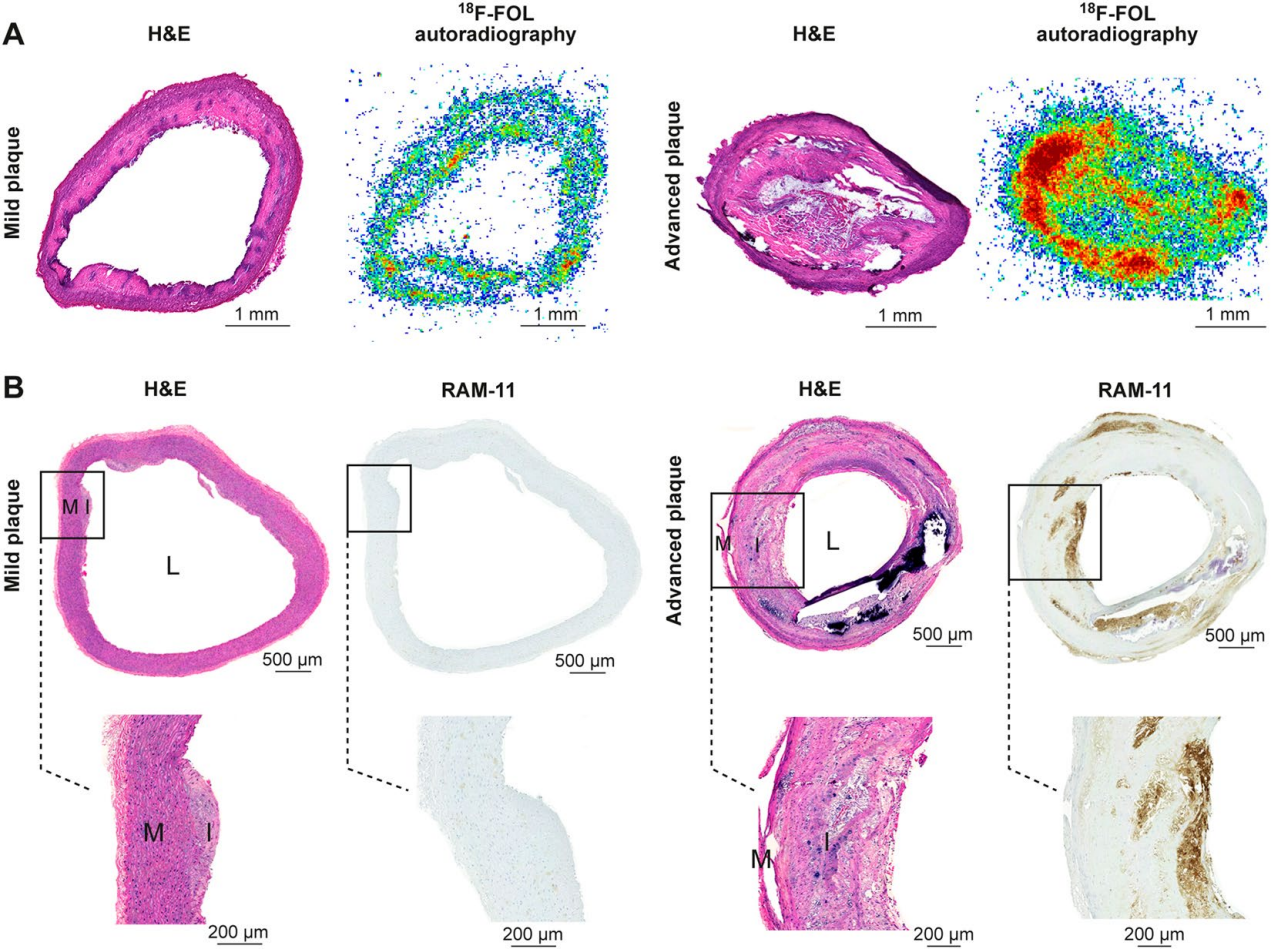
^18^F-FOL uptake, histology and immunohistochemistry of rabbit aorta. Representative (**A**) hematoxylin-eosin (H&E) stained cryosections and ^18^F-FOL *ex vivo* autoradiography images, and (**B**) H&E and RAM-11 immunohistochemistry (macrophages) images of mild intimal thickening and advanced atherosclerosis in rabbit aorta. M = media; I = intima; L = lumen. The images on the bottom show high-power views of the area within the black rectangle on the upper images.

A detailed autoradiographic analysis of cryosections of rabbit aorta revealed a significantly higher uptake of ^18^F-FOL in sections demonstrating advanced atherosclerosis than in sections with mild intimal thickening (41 ± 7.0 *vs*. 17 ± 2.7 PSL/mm^2^, respectively, *P* = 0.0028; Fig. 7A). *In vitro* blocking with folate glucosamine reduced ^18^F-FOL binding in rabbit aorta cryosections by 82% ± 15%.

According to hematoxylin-eosin (H&E) and anti-RAM-11 antibody (macrophages) staining, the aortic segments within the denudated part contained large fibroatheroma-type atherosclerotic lesions. The non-denudated aorta showed some small fibroatheroma-type atherosclerotic lesions in some segments, whereas other segments showed only mild intimal thickening. The infiltration of RAM-11-positive macrophages was widespread in the fibroatheroma-type plaques, and was observed within both the core and fibrous cap. Only limited RAM-11-positive staining was observed in the areas of intimal thickening (Fig. 7B).

## Discussion

^18^F-FOL is a novel tracer for *in vivo* PET imaging of atherosclerotic plaques. Although expression of FR is not an exclusive feature of macrophages, the features of easy radiolabeling, rapid clearance from blood circulation and FR-negative tissues, low uptake in myocardium, and association of high uptake with macrophages in advanced atherosclerotic lesions make ^18^F-FOL a potential tool for imaging of vascular inflammation in atherosclerosis.

Several FR targeting radiotracers have been developed. These have seen slight alterations to their chemistry, with the aim of improving their selectivity, bioavailability, and pharmacokinetic properties^7–11^. Although these other folate-based tracers have accumulated in FR-positive tissues and cells, some non-specific binding to FR-negative tissues has been noticed. For example, ^18^F-fluorophenylfolate has shown high uptake in the intestine (even higher than in kidneys), and in a rodent inflammatory paw model, significant accumulation also occurred in the stomach, bone, liver, and heart^14^. Currently, two folate-based radioconjugates, ^111^In-labeled diethylenetriaminepentaacetic acid (DTPA)-conjugated folate and ^99m^Tc-EC20, have already been tested for *in vivo* SPECT imaging in patients with different cancers^15,16^. Both of the folate agents appeared to be safe and well tolerated, and both exhibited rapid target-tissue uptake and non-target-tissue clearance with urinary excretion. Uptake of ^111^In/^99m^Tc-folates in the liver, lungs, and myocardium was low, which is advantageous for imaging of atherosclerosis. In general, PET imaging can offer several advantages over SPECT imaging, including higher sensitivity, better quantitation, and sharper spatial resolution in a clinical setting^17^. These are important for imaging of small targets, such as atherosclerotic lesions.

Previously, two folate-based SPECT imaging agents, ^99m^Tc-EC20 and ^111^In-EC0800, have shown promising results for *in vivo* detection of atherosclerotic lesions in mice^8,9^. Additionally, Jager and co-workers reported that in tissue sections of atherosclerotic human carotid arteries, fluorescein isothiocyanate-conjugated folate (folate-FITC) targeted FR-β-positive macrophages, and was associated with hypoxia in human atherosclerotic lesions^10^. Later, Müller and co-workers showed that an ^18^F-labeled folate derivative, 3′-aza-2′-^18^F-fluorofolic acid, also detects FR-β-positive macrophages in human atherosclerotic plaques *in vitro*^11^. However, the *in vivo* imaging of atherosclerotic plaque inflammation with a folate-based PET tracer and a comparison with ^18^F-FDG have not been reported, so we therefore performed a direct comparison in both mice and rabbit models. For this part of the study, we used two models of atherosclerosis: (1) hypercholesterolemic LDLR^−/−^ApoB^100/100^ mice that spontaneously develop atherosclerotic lesions in the aorta, which can be accelerated by a high-fat diet^18,19^, and (2) surgical injury accelerated atherosclerosis in Watanabe heritable hyperlipidemic rabbits^20^. In comparison with ^18^F-FDG, our new ^18^F-FOL tracer has lower myocardial uptake and is cleared more rapidly from circulating blood. Recent studies using ^18^F-NaF as a marker of microcalcification in atherosclerosis^21^ and a somatostatin receptor–binding PET tracer ^68^Ga-DOTATATE as a marker of macrophages^22^, have shown that tracers with low retention in the myocardium can provide superior imaging in the coronary arteries as compared with ^18^F-FDG. Furthermore, ^18^F-fluorocholine^23,24^ and ^11^C-PK11195^25^ have been tested for imaging atherosclerotic inflammation in the carotid arteries as alternatives for ^18^F-FDG with potentially less signal from the myocardium. In atherosclerotic mice, the average plaque-to-healthy vessel wall ratio of ^18^F-FOL was 2.6 ± 0.58, which is higher than the ratios of 2.1 ± 0.16 and 2.1 ± 0.5, which we recently reported for ^18^F-FDG and ^68^Ga-DOTATATE, respectively^19,24,26^. The high TBR of ^18^F-FOL was due to both the higher target uptake and the lower blood radioactivity in comparison with ^18^F-FDG. As anticipated, we observed the highest radioactivity of ^18^F-FOL in kidneys, which demonstrate the prominent expression of FR^27^. However, as the excretion of the tracer occurs mainly through the kidneys, it is not possible to distinguish the proportion of tracer specifically bound to kidney FR from that being eliminated by this organ.

Al^18^F-based labeling is one of the most amenable radiosynthesis techniques for clinical settings^28,29^. In this study, we successfully labeled folate with a [^18^F]AlF-NOTA chelating system; however, the critical part was correct formulation of ^18^F-FOL. We always observed a minor radioactive HPLC peak (retention time approximately 6 min) in addition to the end product ^18^F-FOL (Fig. S5A). Under some conditions (e.g., at low pH, high radioactivity concentration), the proportion of the side product increased with time. This may be an indication of radioactivity-induced instability. In order to stabilize ^18^F-FOL, we formulated the tracer in PBS (pH 7.4) in the presence of 8% ethanol and 4–7% polypropylene glycol, keeping the radioactivity concentration under 400 MBq/mL at the end of synthesis. Under these optimized conditions, ^18^F-FOL was stable for at least 4 hours at room temperature (a longer time was not tested). We chose to use polypropylene glycol as it is routinely used in radiopharmaceuticals in our hospital, and is very effective for prevention of radioactivity-induced tracer instability^30^.

A limitation of the current study was that we did not test how the signal detected with folate imaging changes when plaques stabilize, or the effects of statin treatment on the expression of FR on macrophages. Furthermore, the expression of FR and the binding of the radiotracer were not compared between unstable vs. stable carotid plaques. Neither did we provide co- or adjacent comparisons of ^18^F-FOL uptake and FR immunostaining in healthy human aorta or in atherosclerotic plaques from the rabbit model, because we had limited access to normal human tissue and did not find rabbit recognizing anti-FR antibody with optimal immunohistochemical staining of cryosections. We observed higher aorta/blood ratio of ^18^F-FOL in C57BL/6N control mice than in LDLR^−/−^ApoB^100/100^ atherosclerotic mice, and higher ^18^F-FOL radioactivity in the aorta of control mice than in LDLR^−/−^ApoB^100/100^–blocked mice. This may be due to non-specific binding, the presence of FR in non-atherosclerotic aorta or differences in tracer biokinetics between the mouse strains. We hypothesize that our C57BL/6N mice had low aortic expression of FR (FR-positive macrophages) sensitively detectable by the ^18^F-FOL whereas pre-injection of high dose of folate glucosamine had almost completely occupied FRs and blocked ^18^F-FOL binding in the atherosclerotic aorta. According to Müller and co-workers, the FR level in human normal aorta is very low, which supports clinical translation of ^18^F-FOL^11^. It should also be highlighted that the ^18^F-FOL PET imaging in rabbits used a 90-min dynamic acquisition, but the ^18^F-FDG acquisition started 160 min post-injection. This difference may have generated differences in SUV values between ^18^F-FOL and ^18^F-FDG PET measurements.

In conclusion, we demonstrated the feasibility of folate-based PET imaging of inflamed atherosclerotic plaques *in vivo*, using the new imaging agent ^18^F-FOL. Molecular imaging of disease activity in atherosclerosis is a potential approach to evaluate new therapies and even assess the risk of cardiovascular disease and its complications in at risk individuals^3^. Although mainly M2-like macrophage subtypes express FR-β in atherosclerotic lesions^12^, it is too early to say what pathophysiological processes ^18^F-FOL uptake reflects in atherosclerosis. Given the potential translatability of the novel tracer for imaging patients, future studies are warranted to test whether the tracer is suitable for detecting active atherosclerosis in patients.

## Methods

### Production of ^18^F-FOL and ^19^F-FOL

We produced the ^18^F-FOL alias [^18^F]AlF-NOTA-folate according to known procedures^13,31^. Briefly, [^18^F]-fluoride (3.2–3.4 GBq) in physiological saline (50 µL) was added to a reaction vessel containing sodium acetate buffer (pH 4.0, 1 M, 25 µL) and AlCl_3_ (2 mM). The reaction mixture was kept at room temperature for 3 minutes. NOTA-folate (250 µg) in water (50 µL) and acetonitrile (125 µL) was then added, and the reaction vessel was heated at 100 °C for 15 min, followed by dilution of the reaction mixture with water containing 0.2% formic acid (1 mL). In the next step, we performed a high-performance liquid chromatography (HPLC) purification with a semi-preparative C18 column (Jupiter 250 × 10 mm, Phenomenex Inc., Torrance, CA, USA) with both UV (254 nm) and radioactivity detection. Solvent A was water containing 0.1% formic acid and solvent B was acetonitrile containing 0.1% formic acid. The elution was programmed as a gradient from 8% B to 21% B over 20 min, with a flow rate of 4 mL/min. A solution of potassium bicarbonate (KHCO_3_) in water (1 M) was added to adjust the collected HPLC fraction to pH 5.5, and the solvents were evaporated off. The residue was then formulated in phosphate-buffered saline (PBS, 1–3 mL) containing 8% ethanol and 4–7% polypropylene glycol. The radioactivity concentration in the end product bottle was <400 MBq/mL at the end of synthesis. After sterile filtration (0.22 µm, Millipore) into the end product bottle, the tracer was ready for the PET studies. The total synthesis time was 80–95 min, commencing from the end of bombardment. We performed HPLC analysis of the end product on an analytical C18 column (Jupiter 250 × 4.6 mm, Phenomenex Inc., Torrance, CA, USA) with both UV (254 nm) and radioactivity detection (Fig. S5A,B). Solvent A was water containing 0.1% trifluoroacetic acid (TFA) and solvent B was acetonitrile containing 0.1% TFA. The elution was programmed as a gradient from 10% B to 25% B over 15 min, with a flow rate of 1 mL/min. To confirm the identity of ^18^F-FOL, the corresponding “cold” counterpart ^19^F-FOL was used as a reference (Fig. S5C). In addition, the identity of ^18^F-FOL was further confirmed with the analyses of a sample spiked with ^19^F-FOL (Fig. S5D,E). Under the same HPLC conditions, the retention time of precursor NOTA-folate (Fig. S5F) was clearly different from ^18^F-FOL.

For preparation of ^19^F-FOL, NOTA-folate (4.2 mg, 4.9 µmol), aluminum chloride hexahydrate (AlCl_3_·6H_2_O; 5.3 mg, 20 µmol) and sodium fluoride (NaF; 3.7 mg, 90 µmol) were dissolved in 0.1 mL of 0.1 M sodium acetate pH 4.0, resulting in a clear solution. The mixture was heated in a heating block at 105 °C for 15 min, leading to a suspension, which was diluted to 0.3 mL with Milli-Q water and adjusted to pH 7. The white solid was removed by centrifugation and then filtration, affording a clear and yellow solution. The above mixture was submitted to HPLC for further purification: Sunfire C18 column (5 μm, 10 × 250 mm; Waters Corporation, Milford, MA, USA), A = 0.1% acetic acid in water, B = ethanol, column equilibration 8% B for 10 min, gradient 12% B to 40% B in 25 min, flow rate 2 mL/min. The fractions were combined and concentrated under vacuum. The resulting residue was dissolved in 1 mL Milli-Q water and dispensed into small vials, which served as the standard reference. For the high-resolution mass spectrometry (MS) analysis of ^19^F-FOL, the samples were loaded into an offline nanospray emitter (Thermo Scientific, Waltham, MA, USA) and analyzed in positive electrospray ionization (ESI)-MS mode, using an orbitrap mass analyzer in the LTQ Orbitrap Velos Pro mass spectrometer (Thermo Scientific) equipped with a nano-electrospray ionization source. MS data were acquired using Xcalibur 3.0 software (Thermo Scientific). Before the analysis, the mass spectrometer was calibrated with an in-house mixed Calmix LTQ ESI Positive Ion Calibration Solution. Data were analyzed using Xcalibur 4.0 software (Thermo Scientific; Fig. S6).

To evaluate ^18^F-FOL stability in the injectable formulation, we kept the end product at room temperature and took samples for radio-HPLC analysis at time intervals of up to 4 hours. For the quality control of ^18^F-FOL, we used the same HPLC protocols as described above.

To determine the lipophilicity of ^18^F-FOL, approximately 8 kBq of ^18^F-FOL in 500 μL PBS (pH 7.4) was added to 500 μL of 1-octanol in an Eppendorf tube. Mixtures were then vortexed for 3 min and centrifuged (12,000 × *g* for 6 min at room temperature). Following this, 100 μL aliquots of both PBS and 1-octanol layers were counted using a γ-counter (1480 Wizard 3”; Perkin Elmer/Wallac, Turku, Finland). We performed measurements in triplicate, and calculated the octanol-PBS distribution coefficient log *D* as log_10_ (counts in octanol/counts in PBS).

To evaluate the *in vivo* stability and plasma protein binding of ^18^F-FOL, blood samples from six atherosclerotic mice and four rabbits were drawn at 5–60 min post-injection of ^18^F-FOL. Plasma was separated by centrifugation (2,100 × *g* for 4 min at 4 °C) and plasma proteins were separated by adding an equal volume of acetonitrile followed by centrifugation as above. The radioactivity concentration in the protein precipitate and supernatant was then measured (1480 Wizard 3″; Perkin Elmer/Wallac, Turku, Finland). The plasma supernatant was then filtered through a 0.45 µm Minispike filter (Waters Corporation, USA) for further analysis by HPLC. A semi-preparative C18 column (Jupiter 250 × 10 mm, Phenomenex Inc., Torrance, CA, USA) was used for HPLC analysis of mouse plasma samples with both UV (254 nm) and radioactivity detection. Solvent A was water containing 0.1% TFA and solvent B was acetonitrile containing 0.1% TFA. The elution was programmed as a gradient from 7% B to 22% B over 15 min, with a flow rate of 5 mL/min.

### Microscopic equipment

All photomicroscopic images were taken by scanning slides with a digital slide scanner (Pannoramic 250 Flash, 3DHistec Ltd., Budapest, Hungary), with brightfield illumination by a 6 W strobe light, 45x magnification by default, and resolutions of 26x, 41x, and 52x, in an acceptable slide format of 25 × 75 mm, 0.9–1.2 mm thickness. We used Pannoramic viewer software (version 1.15.2) to capture images.

### Human carotid artery samples

We conducted this study according to the declaration of Helsinki, and the study protocol was approved by the ethics committee of the Hospital District of Southwest Finland. All study patients gave their written informed consent. We obtained carotid endarterectomy samples from five patients (four females and one male; aged 70 ± 10 years) with transient ischemic attack and severe (≥80%) stenosis of the carotid artery. The frozen samples containing atherosclerotic plaque were used for *in vitro* ^18^F-FOL binding study and immunohistochemical staining.

### Experimental animals

Male low-density lipoprotein receptor deficient mice expressing only apolipoprotein B100 were used (LDLR^−/−^ApoB^100/100^, strain #003000, The Jackson Laboratory, Bar Harbor, ME, USA). Prior to imaging experiments, a Western-type diet (0.2% total cholesterol, TD 88137, Harlan Teklad, Harlan Laboratories, Madison, WI, USA) was started at the age of 2 months and maintained for 4 months to induce development of inflamed atherosclerotic lesions as previously described^19^. Eight-month-old C57BL/6N mice fed with regular chow were used as healthy controls.

We also used adult Watanabe heritable hyperlipidemic rabbits with a mutated LDLR gene resulting in high circulating cholesterol levels. When the rabbits were at the age of 6 months, a trained professional (J.H.) performed balloon endothelial denudation of the abdominal aorta^20^. Animals were anesthetized with a mixture of ketamine (15 mg/kg, Ketalar, Pfizer, New York, NY, USA) and medetomidine (0.3 mg/kg, Domitor, Orion Pharma, Espoo, Finland). A 3F Fogarty embolectomy balloon catheter (120403F, Edwards Lifesciences, Irvine, CA, USA) was inserted via the right femoral artery and advanced proximally for 20 cm up to the lower thoracic aorta. The catheter was inflated with 0.6 mL of air and pulled down to the level of the aortic bifurcation for endothelial denudation, with the procedure being repeated three times. After removal of the catheter, the femoral artery was ligated and the wound was closed in layers with resorbable suture. Rabbits were returned to their cages after recovery from anesthesia. Rabbits were maintained on a normal diet for the whole study and used for imaging studies at the age of 45 months. More detailed characteristics of the study animals are given in Table 1.

All animals were housed in an animal facility throughout the study, under standard conditions with lights on from 6.00 a.m. to 6.00 p.m., and access to water and food *ad libitum*. All animal experiments were approved by the national Animal Experiment Board in Finland, and the Regional State Administrative Agency for Southern or Eastern Finland, and were carried out in compliance with the European Union directive.

### *In vitro*^18^F-FOL studies

To determine *in vitro* binding of ^18^F-FOL in atherosclerotic plaques, we used human (24 × 7 µm carotid cryosections from five patients), mouse (30 × 8 µm aorta cryosections from four atherosclerotic mice), and rabbit (46 × 20 µm aorta plaque cryosections from four Watanabe rabbits) samples. Firstly, the tissue cryosections were pre-incubated in PBS (pH 7.4) at room temperature for 15 min, and then with 0.2 nM ^18^F-FOL in PBS with or without a 100-fold molar excess of folate glucosamine (C_25_H_30_N_8_O_10_, molecular weight 602.56). After incubation, slides were washed twice with ice-cold PBS for 1 min, dipped in ice-cold distilled water, briefly dried with a hair dryer, and then apposed to an imaging plate (Fuji Imaging Plate BAS-TR2025, Fuji Photo Film Co., Ltd., Tokyo, Japan). After an exposure time of 3.5 to 4 hours, the imaging plates were scanned using Fuji Analyzer BAS-5000 (Fuji Tokyo, Japan; internal resolution of 25 μm). For quantitative analysis of digital autoradiographs, we used Tina 2.1 software (Raytest Isotopemessgeräte, GmbH, Straubenhardt, Germany).

To study the binding of ^18^F-FOL on macrophages, peripheral blood mononuclear cells (PBMCs) from buffy coats were collected using Ficoll centrifugation method. Monocytes were enriched using magnetic-activated cell sorting (MACS) positive selection kit (Monocyte isolation kit with CD14 MicroBeads; Miltenyi Biotec, Bergisch Gladbach, Germany). MACS-purified monocytes were cultured in 12 well plate (1 × 10^6^ cells/well) in the Iscove’s Modified Dulbecco’s medium (IMDM containing 2% AB serum and 2 mmol l-glutamine; Gibco, Thermo Fisher Scientific, Waltham, MA, USA) with M-CSF (20 ng/mL; Peprotech, London, UK) for 6 days at 37 °C in a CO_2_ incubator to obtain macrophages (at day 3 half of the medium was replaced with fresh medium with M-CSF (20 ng/mL). At day 6, to differentiate macrophages to M1, IFN-γ (50 ng/mL; Peprotech) and LPS (100 ng/mL; Sigma-Aldrich, St. Louis, MO, USA) were added to fresh medium, and to differentiate macrophages to M2, M-CSF (20 ng/mL), IL-4 (10 ng/mL; Peprotech) and IL-10 (10 ng/mL; Peprotech) were added to the fresh medium, and incubated for 2 days at 37 °C in a CO_2_ incubator. At day 8 medium from the wells were removed, and ^18^F-FOL (1 MBq/mL in fresh medium) was added to each well and incubated for 1 hour at 37 °C in a CO_2_ incubator. After incubation cells were rinsed with PBS (2 × 1 mL) to remove any unbound radioactive materials. Then, 1% sodium dodecyl sulfate in PBS (1 mL) was added to each well and solubilized cells were collected into individual test tubes^13^. Bound ^18^F-FOL was measured in a γ- counter (1480 Wizard 3″; Perkin Elmer/Wallac, Turku, Finland).

To evaluate the polarization of macrophages to M1 and M2, cells were harvested on day 8. After pre-blocking with human immunoglobulin (Ig 100 μg/mL; KIOVIG, Baxter, Vienna, Austria) cells were incubated with Alexa Fluor 488-conjugated anti-human-CD206 antibody (mouse IgG1; BioLegend, San Diego, CA, USA) for cell surface staining or with isotype control (mouse IgG1; BD Biosciences, New Jersey, NJ, USA). For CD68 staining, cells were permeabilized (15 s in ice-cold acetone), blocked and incubated with Alexa Fluor 488-conugated anti-human-CD68 antibody (mouse IgG2a; BioLegend) or with isotype control (mouse IgG2a; BD Biosciences). Finally, cells were fixed using paraformaldehyde and analyzed using fluorescence-activated cell sorting (FACS) with Fortessa flow cytometer (BD Biosiences) and Flowing software (Turku Center of Biotechnology, Turku, Finland).

### PET/CT and image analysis

In the mice studies, animals were fasted for 4 hours before the PET/CT imaging. Mice were then anesthetized using isoflurane (3–4% induction, 1–2% maintenance), placed onto the bed of a dedicated small animal PET/CT (Inveon Multimodality, Siemens Medical Solutions, Knoxville, TN, USA) and administered with ^18^F-FDG (10 ± 0.3 MBq; 250 ± 32 MBq/kg i.v.) via a tail vein cannula. Mice were imaged again on the following day with ^18^F-FOL (11 ± 0.7 MBq; 280 ± 50 MBq/kg i.v.). For blocking experiments, a 100-fold molar excess of folate glucosamine (25–30 µL) was i.v. injected a minute before ^18^F-FOL. Following a CT for attenuation correction, emission data were acquired in a list mode for 90 minutes. Immediately after PET, an iodinated contrast agent (100 μL of eXIATM160XL, Binitio Biomedical Inc., Ottawa, ON, Canada) was i.v. injected, and a high-resolution CT was performed for anatomical reference. The PET data were iteratively reconstructed with an ordered-subsets expectation maximization 2D algorithm (OSEM2D with two iterations) into 5 × 60 s, 3 × 300 s, 1 × 600 s, and 2 × 1,800 s time frames (matrix size: 128 × 128 × 159, pixel size: 0.776 × 0.776 × 0.796 mm). Vascular contrast-enhanced CT images were reconstructed with a Feldkamp based algorithm (matrix size: 768 × 768 × 923, pixel size: 0.094 × 0.094 × 0.094 mm). Co-registration of PET and CT images was automatic, and confirmed visually on the basis of anatomical landmarks. To analyze PET/CT images, we used the free Carimas 2.9 software (Turku PET Center, Turku, Finland, www.turkupetcentre.fi/carimas/). We defined regions of interest (ROIs) for the aortic arch, vena cava (representing blood), and myocardium in the coronal PET/CT images, using the contrast-enhanced CT as an anatomical reference. The transaxial and sagittal views were used to ensure the correct ROI placement. We expressed results as standardized uptake values (SUVs), normalized for the injected radioactivity dose and animal body weight, and as maximum target-to-background ratios (TBR) calculated as SUV_max, aortic arch_/SUV_mean, blood_. For both ^18^F-FDG and ^18^F-FOL studies, we defined the blood ROI in the same area of the vena cava. In addition to *in vivo* imaging, we performed *ex vivo* ^18^F-FOL PET imaging for excised mice aorta. After preparation of aortas, they were placed on a Petri dish that was covered with ultrasound gel to avoid dehydration, and PET data were acquired for 30 minutes, followed by reconstruction using the OSEM3D algorithm (2 iterations; matrix size: 128 × 128 × 159, pixel size: 0.776 × 0.776 × 0.796 mm).

In the rabbit studies, animals were fasted for at least 4 hours prior to the scans. They were sedated and anesthetized with subcutaneous injection of medetomidine hydrochloride (Domitor, Orion Pharma; dose, 0.1 mg/kg) and ketamine (Ketalar, Pfizer; dose, 30 mg/kg), and a urinary catheter was inserted to minimize accumulation of radioactivity in the bladder. The rabbits were placed in a supine position in a clinical PET/CT scanner (Discovery VCT, General Electric Medical Systems, GEMS, Milwaukee, WI, USA). All of the rabbit PET studies started with a low-dose CT for attenuation correction. The tube voltage was 80 or 120 kV, and the tube current was 10−80 mA with tube current modulation. The slice thickness was 3.75 mm. On the first imaging day, ^18^F-FDG (140 ± 29 MBq; 45 ± 1.5 MBq/kg) was i.v. administered via a marginal ear vein cannula and the PET acquisition was begun 160 min post-injection. Two bed positions were imaged (10 minutes for each 15.7 cm bed position). The total imaging time was therefore 20 minutes. On the following day, the same rabbits were re-imaged with ^18^F-FOL (130 ± 37 MBq; 41 ± 3.7 MBq/kg) and a dynamic PET study was performed with two bed positions. The acquisition times were 4 × 15 s, 6 × 30 s, 6 × 60 s, and 10 × 120 s. Bed positions were imaged in an interleaved manner and the total scanning time was 90 minutes. After PET imaging, CT was performed with a tube voltage of 100 kV and a current of 450 mA. The CT field-of-view covered the area from the aortic arch to the bifurcation, and the slice thickness was 0.625 mm. After scanning, PET data were reconstructed with a three-dimensional iterative algorithm (VUE point) with 28 subsets, 2 iterations, a standard z axis filter, and 2.00 mm full width at half maximum post-filtering. Attenuation correction was performed using low-dose CT. The PET image matrix size was 256 × 256, the pixel size was 1.37 × 1.37 mm, and the field-of-view was 35 cm. We performed analysis of PET/CT images as described above. We divided the aorta into 2 cm segments and determined the maximum SUV in each of them. The mean SUV in the blood was measured from the inferior vena cava to calculate maximum TBRs (SUV_max_, _aorta_/SUV_mean, blood_). We carefully identified the anatomical landmarks (e.g., renal arteries) from CT images in order to match up the defined 2 cm aorta segments to the excised samples used for *ex vivo* gamma counting and autoradiography.

All PET/CT images were exported from Carimas 2.9 to Adobe Photoshop CS5 with a resolution of 1,648 × 948 pixels for preparation of Figs 3 and 5.

### *Ex vivo* biodistribution of ^18^F-FOL

Two hours after injection of ^18^F-FOL, mice and rabbits were euthanized. Blood samples were drawn from mice by cardiac puncture under deep isoflurane anesthesia, and the mice were then sacrificed with cervical dislocation. The aorta and other tissues were excised and weighed, and their radioactivity was measured with a γ-counter (Triathler 3′′, Hidex, Turku, Finland). Blood samples were drawn from rabbits by cardiac puncture under deep medetomidine hydrochloride/ketamine anesthesia, and the rabbits were then sacrificed with an overdose of pentobarbital (Mebunat vet 60 mg/mL, Orion Pharma, Espoo, Finland). From each rabbit, the aorta from the aortic arch to the iliac artery bifurcation was prepared and cut into 2 cm segments. These were weighed, and the radioactivities of each aorta segment, blood, and various tissue samples were measured using a γ-counter (1480 Wizard 3′′; Perkin Elmer/Wallac, Turku, Finland). To determine the *ex vivo* biodistribution, we normalized the radioactivity values for injected radioactivity dose, decay of ^18^F, and weight of tissue sample. In mice, we compensated for the radioactivity remaining in the tail. The *ex vivo* biodistribution results were expressed as a percentage of the injected radioactivity dose per gram of tissue (%ID/g).

### Autoradiography

After *ex vivo* gamma counting of excised tissues, we studied the aortic distribution of ^18^F-FOL in more detail using autoradiography as described earlier^19^. Whole excised aorta from the mice was used, as well as two segments of abdominal aorta and one segment of thoracic aorta from the rabbits. These were chosen on the basis of the ^18^F-FDG uptake seen on the PET/CT images the day before sacrifice. The excised mouse aortas were frozen in dry-ice cooled isopentane and cut into sequential longitudinal cryosections of 20 μm. The rabbit aortas were divided into 3–6 transverse 2 cm segments, frozen, and cut into 20 μm sequential transverse cryosections. The sections were then apposed on an imaging plate (Fuji Imaging Plate BAS-TR2025), and the imaging plates were scanned (Fuji Analyzer BAS-5000) after an exposure time of at least 4 hours. After scanning, sections were stored at −20 °C until being stained with H&E and scanned with a digital slide scanner (Pannoramic 250 Flash).

After a careful superimposition of the digital autoradiographs and H&E images, we determined ^18^F-FOL accumulation in mice in the following ROIs: (1) atherosclerotic plaques (non-calcification, excluding media), (2) normal vessel wall (no lesion formation), and (3) adventitia (mainly adipose tissue around the aorta). In total, 677 ROIs (282 in plaques, 256 in normal vessel wall, and 139 in adventitia) were analyzed from ten atherosclerotic (including two mice used for the blocking study) and six control mice (Table 1). For rabbits, the atherosclerotic plaques were divided into two categories: (1) advanced atherosclerotic plaques (large fibroatheroma lesions mainly observed in denudated abdominal aorta) and (2) mild atherosclerotic plaques (small lesions/intimal thickening observed in non-denudated thoracic aorta) on the basis of their histopathological characteristics seen in H&E, and as assessed by J.M.U.S. and A.S., together with an experienced pathologist, P.S. In total, 322 ROIs (220 in advanced plaques and 102 in mild plaques) were analyzed from four rabbits for ^18^F-FOL accumulation. We were not able to measure radioactivity accumulation in totally healthy vessel wall, as some plaque formation was also seen in non-denudated thoracic aorta due to the genetic background and advanced age of the rabbits.

For autoradiography analysis, we used Tina 2.1 software and expressed ^18^F-FOL uptake as photostimulated luminescence per square millimeter (PSL/mm^2^). The count densities of background radiation were subtracted from the actual ROI data. Additionally, the results for each mouse and rabbit were decay-corrected for injection and exposure time, and normalized for injected radioactivity dose. Finally, we calculated the average ^18^F-FOL uptake ratios between atherosclerotic plaque, normal vessel wall and adventitia from each mouse, and the average PSL/mm^2^ values in advanced and mild plaques from each rabbit. The digital autoradiographs were exported from Tina 2.1 with a resolution of 766 × 1,008 pixels (mice) and 398 × 416 pixels (rabbits), and the H&E images were exported from Pannoramic viewer with a resolution of 4,729 × 3,230 pixels (mice) and 12,516 × 29,356 pixels (rabbits), to Adobe Photoshop CS5 for preparation of Figs 2–4 and 7.

### Characterization of atherosclerotic plaques

We used frozen human carotid samples to investigate co-localization of ^18^F-FOL binding, CD68-positive macrophages, and FR-β. For this purpose, macrophages were identified by incubating 8 µm cryosections with mouse monoclonal anti-human CD68 antibody (mo876; 1:200, PG-M1, Dako, Glostrup, Denmark), as previously described^12^. For FR-β immunohistochemical staining, we used a previously described protocol with minor modifications^12^. Briefly, sections were dried at room temperature and fixed with 4% paraformaldehyde. Sections were first incubated with biotinylated anti-human FR-β antibody (m909, 1:100)^32^, and then with secondary antibody (P0397, 1:200, Dako). A color reaction was developed using 3,3′-diaminobenzidine (Dako), and counterstained using Mayer’s hematoxylin. For quality control of the staining procedure, we stained adjacent sections without primary antibody. Images were exported from Pannoramic Viewer into Adobe Photoshop CS5 at a resolution of 4,309 × 20,239 pixels to create Fig. 2.

Mice hearts were collected and preserved in 10% formalin at room temperature overnight. They were then dehydrated using 70% ethanol, embedded in paraffin, and cut transversely into 6 µm sections at the level of the coronary ostia. Adjacent sections were stained with modified Movat’s pentachrome^19^ to detect histopathologic characteristics, or with anti-mouse Mac-3 antibody (1:1000, BD Biosciences, Franklin Lakes, NJ, USA)^33^ to detect macrophages. Additionally, sections were stained with anti-iNOS (1:200, Abcam, Cambridge, UK)) or anti-MRC-1 (1:500, Abcam, Cambridge, UK) antibodies to detect M1 (pro-inflammatory) and M2 (anti-inflammatory) polarized macrophages, respectively^24^. After staining, the sections were scanned with a slide scanner (Pannoramic 250 Flash). From each section (n = 3/mouse/staining), we outlined the areas of intima and media using the free GNU Image Manipulation Program v. 2.9 (www.gimp.org), and determined the total area as mm^2^ using Image-J software v. 1.46 (National Institutes of Health, Bethesda, MD, USA)^34^. To determine macrophage positive area in the intima, we used the automatic color deconvolution method of Image-J, and expressed results as positive staining area in aortic ostia (%). We used Adobe Photoshop CS5 to convert the aortic ostia and high-power view images (resolutions of 1,685 × 1,642 pixels and 4,232 × 1,502 pixels, respectively) to create Fig. 3. We used the aortic ostia to determine the area of macrophages in each mouse, as it could be easily identified because of the valves, and was therefore the most reliable place to compare plaque deposition between different mice.

Frozen mouse aorta samples were used to investigate co-localization of ^18^F-FOL binding, Mac-3-positive macrophages and FR-β. For macrophage detection, 8 µm cryosections were incubated with anti-mouse Mac-3 antibody (1:1000, BD Biosciences, Franklin Lakes, NJ, USA)^33^. For FR-β immunohistochemical staining, 8 µm cryosections were air-dried at room temperature for 30 min and fixed with 4% paraformaldehyde. Sections were first incubated with rabbit polyclonal anti-FR-β antibody (1:100, Biorbyt Ltd, Cambridge, UK), and then with anti-rabbit secondary antibody (Bright vision Poly-HRP-anti Rb, VWRKDPVR110HRP). A color reaction was developed using 3.3′-diaminobenzidine (Bright-DAB, BS04-110), and counterstained using Mayer’s hematoxylin. Images were exported from Pannoramic Viewer into Adobe Photoshop CS5 at a resolution of 24,993 × 58,723 pixels to create Fig. 4.

On the basis of the ^18^F-FDG uptake seen in the PET/CT, certain rabbit aorta segments were used for autoradiography and the other segments were fixed in 10% formalin, embedded in paraffin, and cut into 6 µm sections. For each aorta segment, adjacent sections were stained with either Movat’s pentachrome or H&E for morphological characterization. To detect rabbit macrophages, we deparaffinized adjacent aorta sections by immersion in xylene. A graded ethanol series was then used for rehydration. Sections were then treated in 0.25% Triton X-100 in PBS, 3% H_2_O_2_, and 3% protease-free bovine serum albumin. Sections were incubated with primary antibody against mouse anti-rabbit RAM-11 (M 0633, 1:1000, Envision, Dako, Carpinteria, CA, USA), followed by secondary anti-mouse antibody (K4001, Envision, Dako, Carpinteria, CA, USA). For detection, 3,3′-diaminobenzidine was used as a chromogen (K3468, Envision, Dako, Carpinteria, CA, USA) and sections were counterstained with Mayer’s hematoxylin. We used GIMP and Image-J software (as described above) to determine the RAM-11 positive area (%) in rabbit plaques, and compared the results with the corresponding ^18^F-FOL and ^18^F-FDG uptake seen in the PET/CT in the same aortic segment. We used Adobe Photoshop CS5 to create Figs 7 and S4.

Additionally, we used Microsoft Excel to create other graphs and exported them into Adobe Photoshop CS5 for preparation of Figs 2–7 and S2, S4.

### Statistical analyses

We presented all values as mean ± SD with two significant figures. To compare groups, we used independent samples *t* tests. For multiple group comparisons, we used a one-way ANOVA with Tukey’s correction. Paired *t* tests were applied for comparisons of uptake between different tissues in the same animals. Associations between continuous variables were tested using general linear mixed models, where animal was used as a random effect to account for within-subject correlation, and classified aorta segments were used as a repeated effect to account for the difference between segments when appropriate. The correlations between continuous variables were assessed using Pearson’s correlation coefficient (r). We checked residuals for justification of the analyses and considered *P* values less than 0.05 to be statistically significant. Statistical analyses were conducted with SPSS Statistics 21 (IBM Corp., Armonk, NY, USA) or with SAS software, version 9.4 (SAS Institute Inc., Cary, NC, USA).

## Electronic supplementary material

Supplemental data set_clean
