# Supplementary material for: Aluminum fluoride-18 labeled folate enables in vivo detection of atherosclerotic plaque inflammation by positron emission tomography
Source: Sci Rep. 2018 Jun 26;8:9720. doi: 10.1038/s41598-018-27618-4 (PMC6018703; doi:10.1038/s41598-018-27618-4)
Supplement: Supplementary file 1 — Supplemental data set_clean [file 41598_2018_27618_MOESM1_ESM.pdf]

Supplemental Material

Aluminum fluoride-18 labeled folate enables *in vivo* detection of atherosclerotic plaque inflammation by positron emission tomography

Johanna M. U. Silvola[1], Xiang-Guo Li[1,2], Jenni Virta[1], Päivi Marjamäki[1], Heidi Liljenbäck[1,3], Jarkko P. Hytönen[4], Miikka Tarkia[1], Virva Saunavaara[5,6], Saija Hurme[7], Senthil Palani[1], Harri Hakovirta[8], Seppo Ylä-Herttuala[4,9], Pekka Saukko[10], Qingshou Chen[11], Philip S. Low[11], Juhani Knuuti[1,2,5], Antti Saraste[1,5,12,13] and Anne Roivainen[1,3,5]*

[1]*Turku PET Centre, University of Turku, Turku, Finland;* [2]*Turku PET Centre, Åbo Akademi University, Turku, Finland;* [3]*Turku Center for Disease Modeling, University of Turku, Turku, Finland;* [4]*A. I. Virtanen Institute for Molecular Sciences, University of Eastern Finland, Kuopio, Finland;* [5]*Turku PET Centre, Turku University Hospital, Turku, Finland;* [6]*Department of Medical Physics, Turku University Hospital, Turku, Finland;* [7]*Department of Biostatistics, University of Turku, Turku, Finland;* [8]*Department of Vascular Surgery, Turku University Hospital, Turku, Finland;* [9]*Science Service Center and Gene Therapy Unit, Kuopio University Hospital. Kuopio, Finland;* [10]*Department of Pathology and Forensic Medicine, University of Turku, Turku, Finland;* [11]*Department of Chemistry, Purdue University, West Lafayette, Indiana, USA;* [12]*Heart Center, Turku University Hospital, Turku, Finland;* [13]*Institute of Clinical Medicine, University of Turku, Turku, Finland*

[*]Correspondence: anne.roivainen@utu.fi

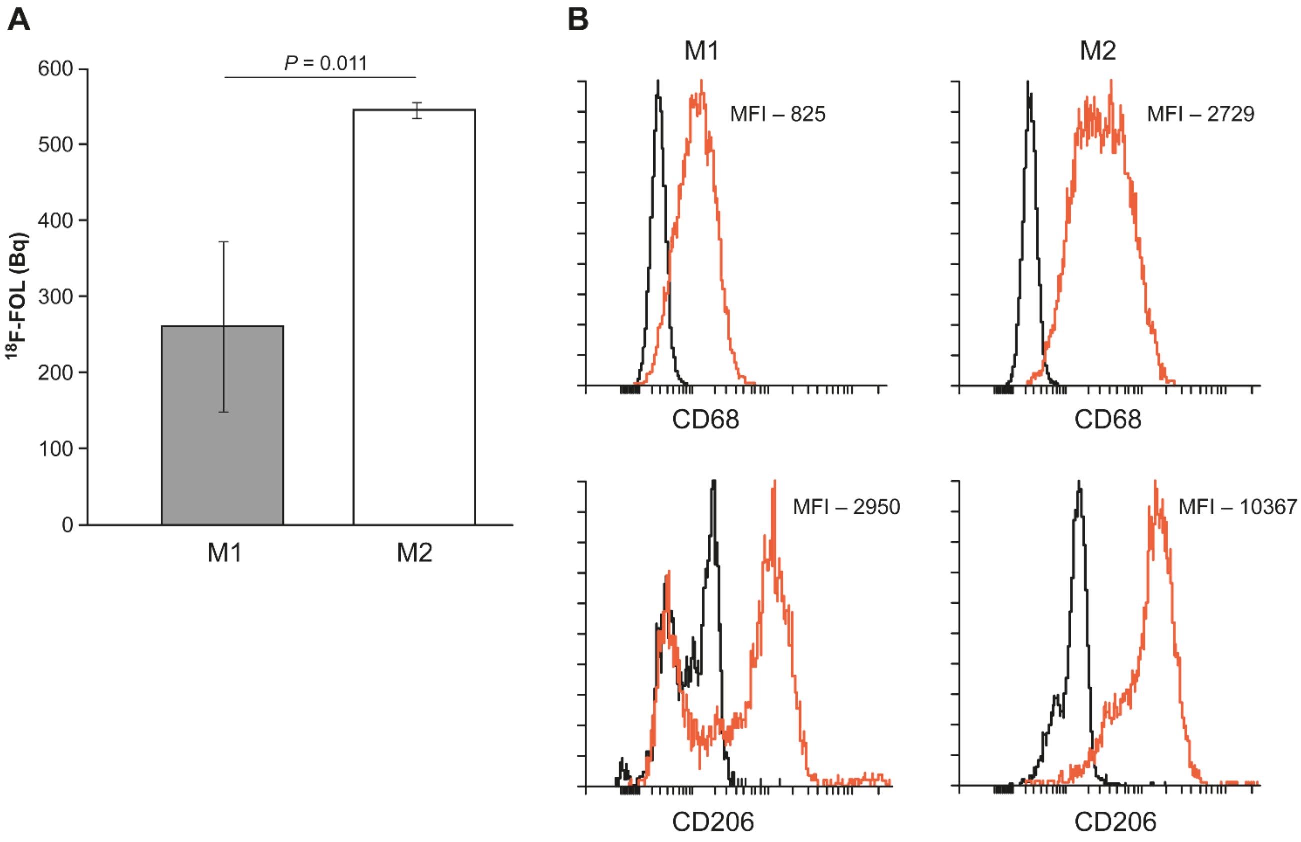

Figure S1. ^{18}F-FOL binds to macrophages derived from human blood monocytes. (A) Binding of ^{18}F-FOL on M1 macrophages (polarized with LPS and IFN-γ) and M2 macrophages (polarized with M-CSF, IL-4 and IL-10). Quantitative data are Becquerel (mean ± standard deviation, n = 3 triplicates). (B) Representative flow cytometric analyses of total CD68 (permeabilized cells) and surface CD206 (macrophage mannose receptor-1 [MRC-1]) from M1 and M2 macrophages. Black histograms are isotype controls and red histograms are for CD68 and CD206. MFI = mean fluorescence intensity.

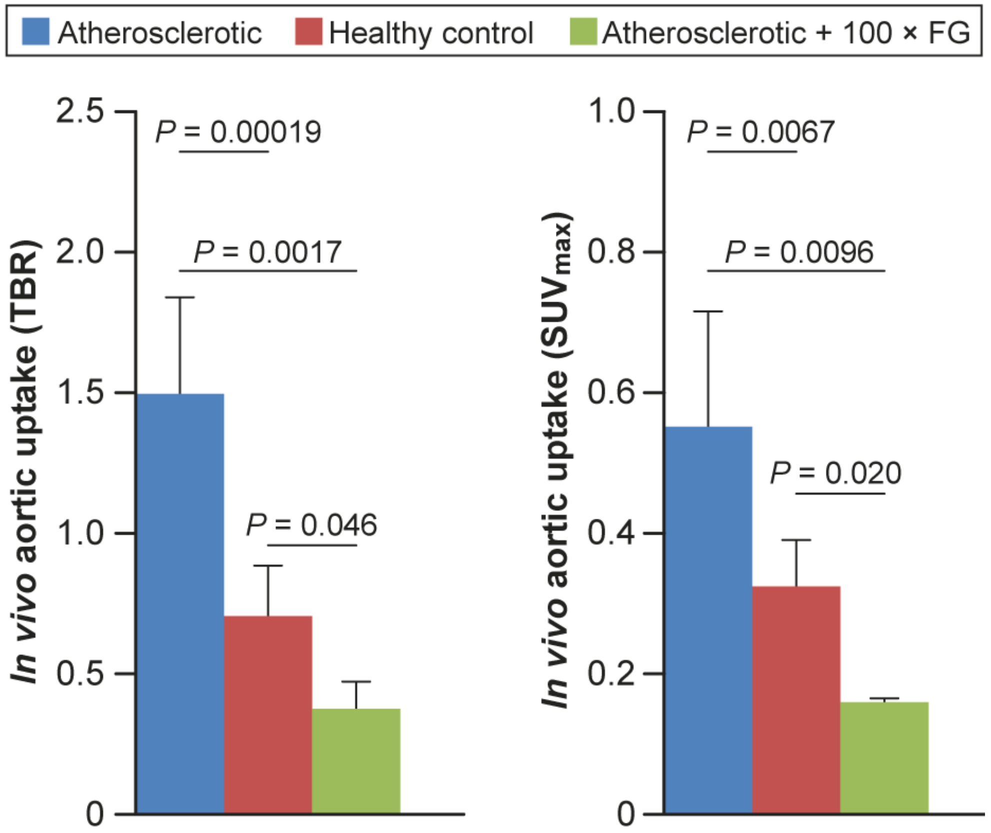

Figure S2. Uptake of ^{18}F-FOL in mice. Uptake of ^{18}F-FOL in aortic arches of atherosclerotic LDLR$^{-/-}$ ApoB$^{100/100}$ (n = 9), healthy C57BL/6N controls (n = 6), and atherosclerotic mice with a 100-fold molar excess of folate glucosamine (FG) (n = 3), as assessed by *in vivo* PET/CT imaging. TBR = maximum target-to-background ratio, $SUV_{max,\ aortic\ arch}/SUV_{mean,\ blood}$. SUV_{max} = maximum standardized uptake value determined at 60–90 min post ^{18}F-FOL injection.

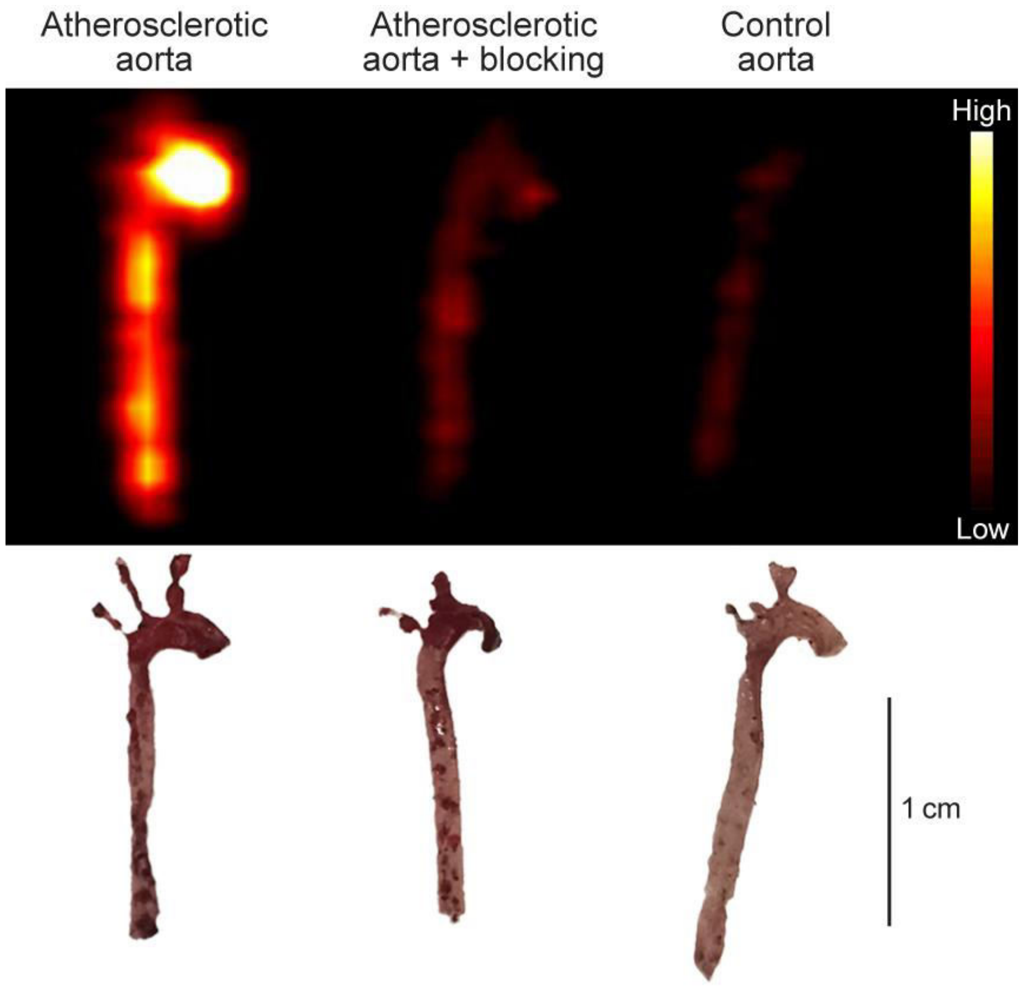

Figure S3. Mouse aorta ^{18}F-FOL PET. *Ex vivo* obtained examples of ^{18}F-FOL PET images (top) and photographs of the aortas stained with Oil-Red-O (bottom) from atherosclerotic LDLR$^{-/-}$ApoB$^{100/100}$ mouse (*n* = 1), atherosclerotic mouse with a 100-fold excess of folate glucosamine (blocking) (*n* = 1), and healthy C57BL/6N control mouse (*n* = 1).

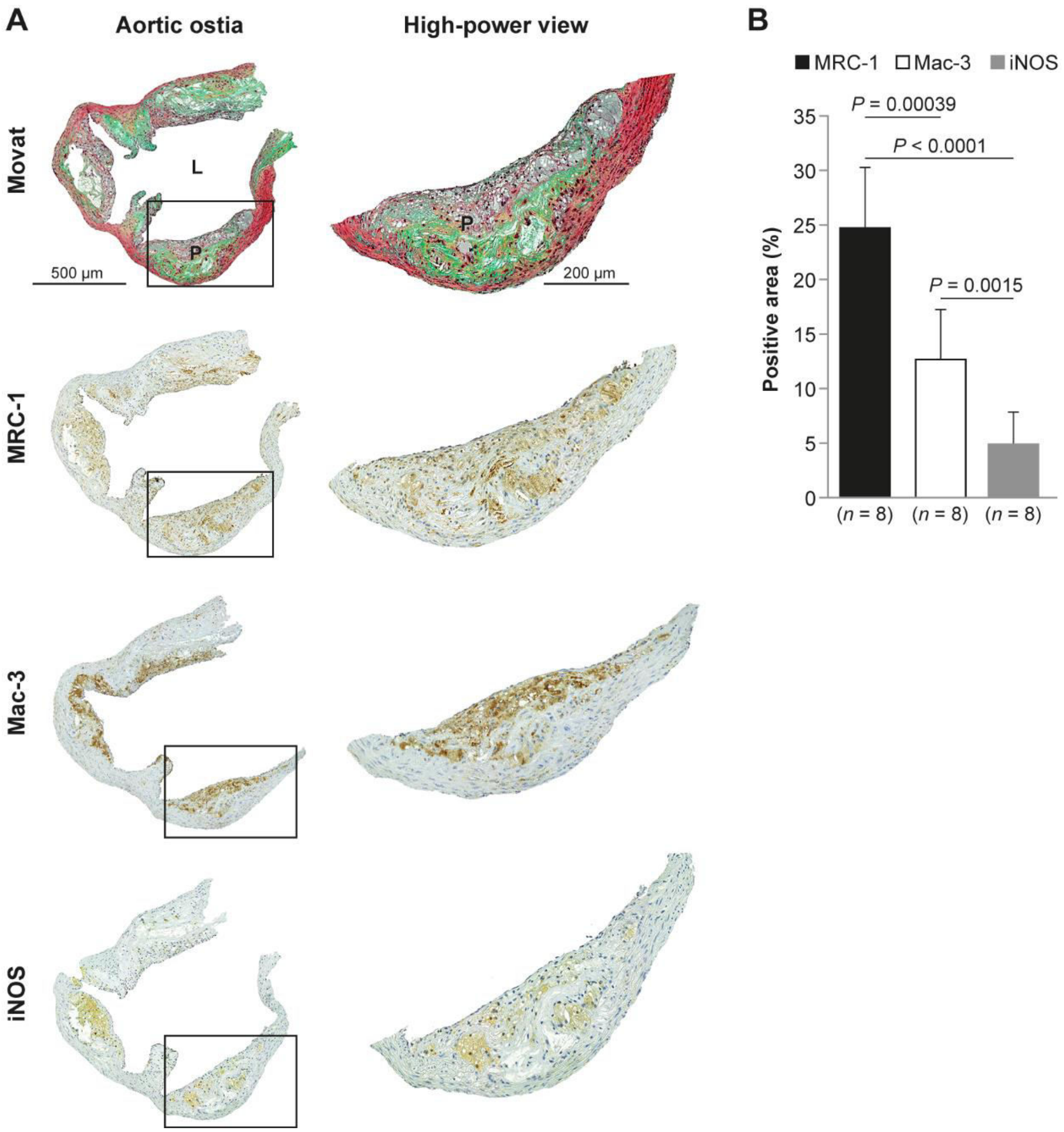

Figure S4. Histology and immunohistochemistry of atherosclerotic lesions in LDLR$^{-/-}$ApoB$^{100/100}$ mice. (A) Representative aortic root sections were stained with Movat's pentachrome (black = nuclei; yellow = collagen, reticular fibers; blue = ground substance, mucin; bright red = fibrin; red = muscle) or with anti-mouse Mac-3 (macrophages), iNOS (M1 polarized macrophages) and MRC-1 (M2 polarized macrophages) immunohistochemistry. The high-power views are of the area within the black rectangle on the left images. Mac-3, iNOS and MRC-1 positive cells appear brown in color. L = lumen; P = plaque; W = healthy vessel wall. Please note that the Movat and Mac-3 images are the same as in

Figure 3D. (B) Quantitative results of immunohistochemistry for the detection of macrophage markers (mean ± SD).

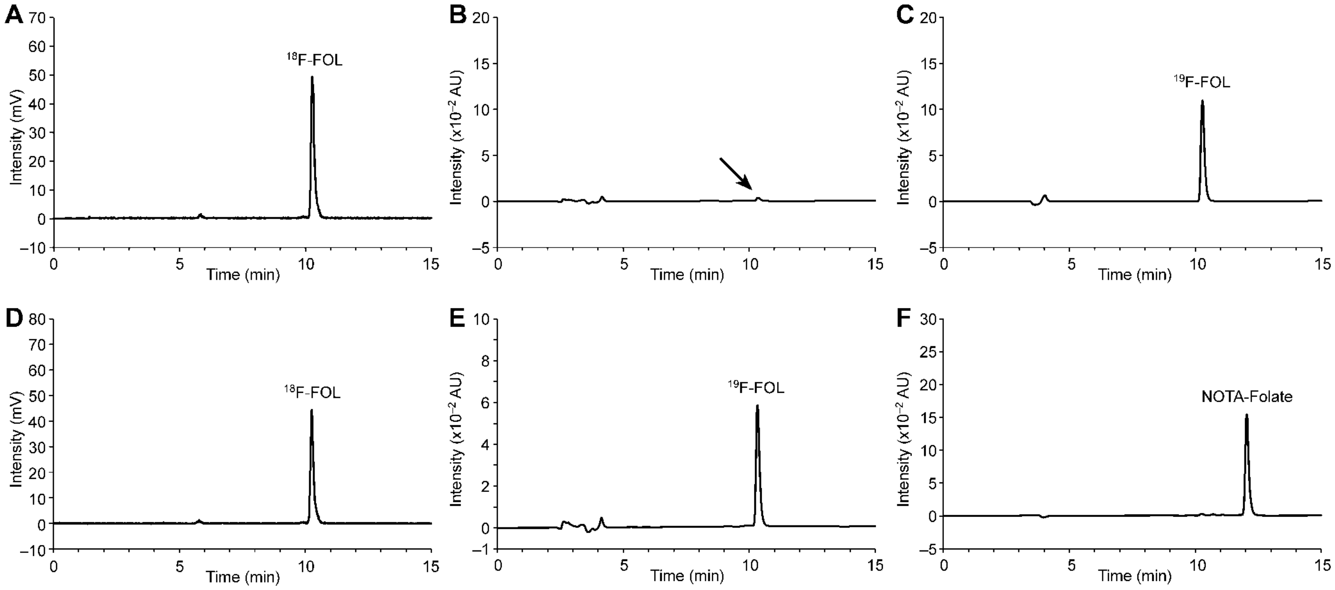

Figure S5. Representative HPLC chromatograms of ^{18}F-FOL, the reference ^{19}F-FOL, and precursor NOTA-folate. (A) Radioactivity and (B) UV detection of ^{18}F-FOL (the UV peak [arrow] was low because of the high specific radioactivity of ^{18}F-FOL), (C) UV detection of ^{19}F-FOL, (D) radioactivity and (E) UV detection of ^{18}F-FOL spiked with ^{19}F-FOL, and (F) UV detection of NOTA-folate.

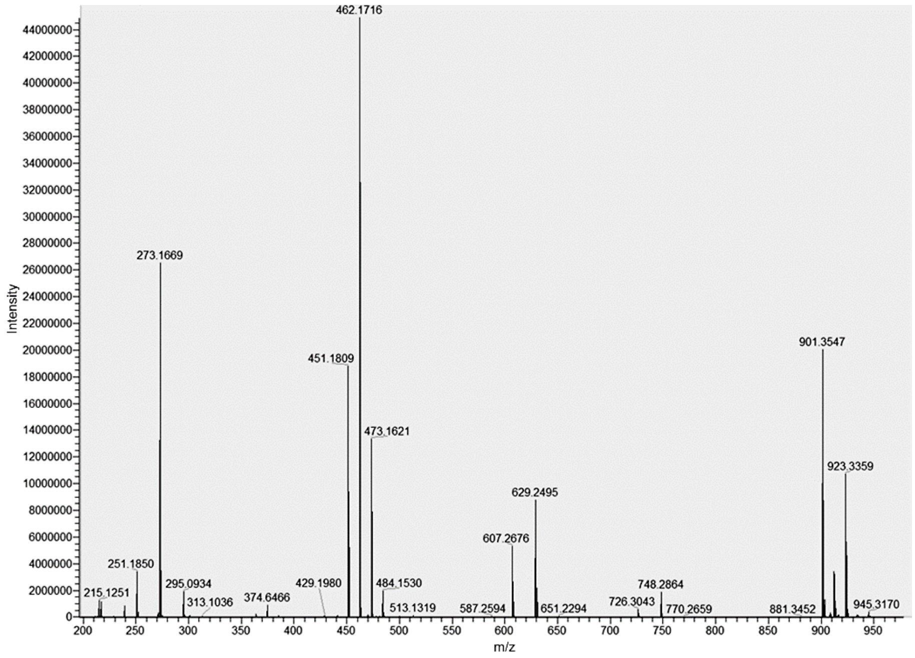

Figure S6. High-resolution mass spectrometry analysis of ^{19}F-FOL. The theoretical monoisotopic mass for ion $[M+H]^+$ $C_{37}H_{51}AlFN_{12}O_{12}$ was 901.3544 and the observed mass was 901.3547.
